# Structural intermediates and directionality of the swiveling motion of Pyruvate Phosphate Dikinase

**DOI:** 10.1038/srep45389

**Published:** 2017-03-30

**Authors:** Alexander Minges, Daniel Ciupka, Christian Winkler, Astrid Höppner, Holger Gohlke, Georg Groth

**Affiliations:** 1 Cluster of Excellence on Plant Sciences (CEPLAS), Institute of Biochemical Plant Physiology, Heinrich Heine University Düsseldorf, 40204 Düsseldorf, Germany; 2 Institute of Pharmaceutical and Medicinal Chemistry, Heinrich Heine University Düsseldorf, 40204 Düsseldorf, Germany

## Abstract

Pyruvate phosphate dikinase (PPDK) is a vital enzyme in cellular energy metabolism catalyzing the ATP- and P*_i_*-dependent formation of phosphoenolpyruvate from pyruvate in C_4_ -plants, but the reverse reaction forming ATP in bacteria and protozoa. The multi-domain enzyme is considered an efficient molecular machine that performs one of the largest single domain movements in proteins. However, a comprehensive understanding of the proposed swiveling domain motion has been limited by not knowing structural intermediates or molecular dynamics of the catalytic process. Here, we present crystal structures of PPDKs from *Flaveria*, a model genus for studying the evolution of C_4_ -enzymes from phylogenetic ancestors. These structures resolve yet unknown conformational intermediates and provide the first detailed view on the large conformational transitions of the protein in the catalytic cycle. Independently performed unrestrained MD simulations and configurational free energy calculations also identified these intermediates. In all, our experimental and computational data reveal strict coupling of the CD swiveling motion to the conformational state of the NBD. Moreover, structural asymmetries and nucleotide binding states in the PPDK dimer support an alternate binding change mechanism for this intriguing bioenergetic enzyme.

Life depends on the light-driven fixation of CO_2_ during photosynthesis. Two major types of photosynthesis have been established during evolution. C_3_ photosynthesis, where primary carboxylation takes place in the Calvin-Benson cycle, is the typical pathway used by most plants. Higher efficiency in light-driven energy conversion is realized in C_4_ plants, where primary carbon fixation is spatially separated from carbon release to the Calvin-Benson cycle. Here, the rate limiting regeneration of the primary CO_2_ acceptor phosphoenolpyruvate (PEP) is catalyzed by the multi-domain protein pyruvate phosphate dikinase (PPDK)^1^. In addition to its essential role in C_4_ plants, PPDK is also found in various bacteria and protozoa, where the enzyme catalyzes the reverse reaction–ATP formation from AMP, pyrophosphate and PEP–during glycolysis^2^. The different directions of the catalytic reaction favored in plants and microorganisms underline that the enzyme is a reversible molecular machine. Bacterial PPDKs are known to form fully active homodimers, while for a number of plant PPDKs the active form was attributed to a homotetramer^3^,^4^,^5^. Ubiquitous distribution and low expression pattern^6^ preclude *in vivo* detection of C_3_-PPDK catalytic products against a background of other pyruvate/PEP converting enzymes such as pyruvate kinase or PEP carboxykinase^2^, leaving the physiological function of PPDK in C_3_ plants largely unknown. Though, cytoplasmic C_3_-PPDK is considered to play a role as secondary glycolytic enzyme based on its abundance in developing rice endosperm^7^.

Previous studies emphasize that PPDK has a modular structure consisting of distinct substrate binding domains for PEP/pyruvate at the C-terminus (PBD) and for nucleotides ATP/AMP at the N-terminus (NBD, aa 1–340) (Supplementary Fig. 1a). The NBD is structured in three subdomains: NBD1 (aa 1–111 and 197–243), NBD2 (aa 112–196), and NBD3 (aa 244–340). A central domain (CD, aa 380–515) catalyzing the phosphoryl group transfer from ATP to pyruvate–or from PEP to AMP–via a phospho-histidine intermediate mediates a catalytic cross-talk between the distant substrate binding domains (Supplementary Fig. 1b)^8^,^9^. Distinct conformational states resolved in crystal structures from maize and the non-plant organisms *Clostridium symbiosum* and *Trypanosoma brucei* suggest that the phosphoryl group transfer in the catalytic cycle is accompanied by a large swiveling motion of the CD from a position next to the NBD to a position facing the PBD (~110° and 45 Å)^8^,^9^,^10^,^11^. Similar single domain conformational re-arrangements have been observed for other proteins or protein assemblies, and domain swiveling is a common mechanism in enzyme catalysis, molecular transport, or electron transfer^12^,^13^,^14^,^15^. Still, the proposed translocation of the PPDK CD reflects one of the largest single domain movements observed in proteins yet. However, detailed insights into the molecular processes during the proposed swiveling motion, in particular on intermediate conformations of the CD and NBD, and potential driving forces behind the motion have remained elusive. Here, we co-crystallized the C_4_-PPDK from *Flaveria trinervia* and the related C_3_-PPDK from *Flaveria pringlei* with their natural product PEP and the substrate analogue 2′-Bromo-2′-deoxy-adenosine-5′-[(*β, γ*)-imido]triphosphate (2′-Br-dAppNHp). Both isoforms share 96% sequence identity–excluding the chloroplast transport peptide–and have a monomeric molecular weight of ~95 kDa. Using X-ray crystallography, we solved for the first time structures of ternary complexes of this multi-domain enzyme. The C_4_-PPDK structures (PDB 5JVJ and 5JVL) resolve intermediates of the NBD opening-closing motion with 5JVL providing the first structural model with bound nucleotide in the NBD. In addition, these structures substantiate that the catalytic histidine in the CD mediating phosphoryl group transfer between the active sites at the N- and C-terminus is positioned in close proximity to the bound PEP molecule. More importantly, the C_3_-PPDK structure from *F. pringlei* (PDB 5JVN) for the first time has trapped an intermediate state of the central domain, shedding light on sequential steps of the swiveling motion. The analysis of essential motions in available crystal structures and unrestrained molecular dynamics simulations reveal coupled motions of the CD and the NBD for non-phosphorylated PPDK. Extensive 1D and 2D potential of mean force (PMF) calculations of the CD motion also reveal the existence of distinct intermediate conformational states, resulting in sawtooth-like free energy profiles that are indicative of a Brownian ratchet mechanism biasing random thermal fluctuations. Furthermore, they suggest a tilting of the configurational free energy profiles depending on the binding state of the NBD and the phosphorylation state of the CD.

## Results and Discussion

### Overall structure of *Flaveria* PPDKs

**Figure 1:**
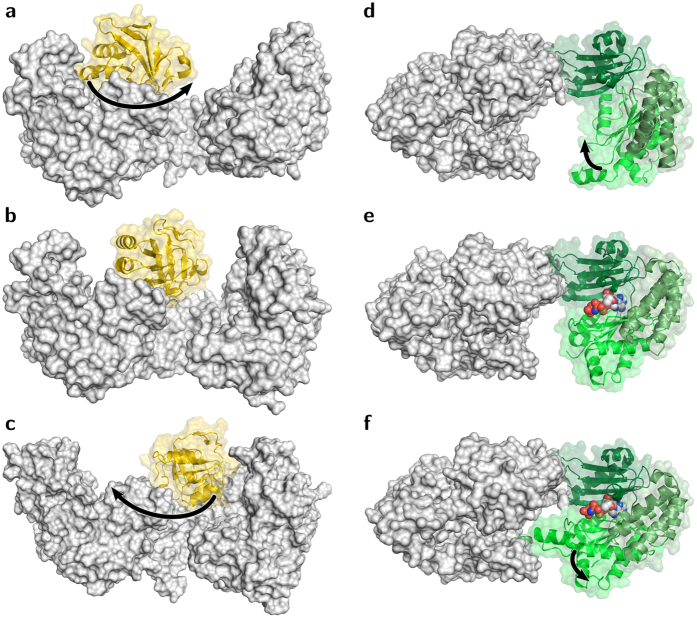
Movement of the PPDK domains. (**a–c**) Movement of the CD (yellow). (**a**) *Ft*PPDK in PBD-facing conformation (PDB 5JVL/C). (**b**) *Fp*PPDK (PDB 5JVN) with CD in an intermediate position. (**c**) *Tb*PPDK (PDB 2X0S)^19^ with CD in NBD-facing conformation. (**d-f)** Movement of the NBD (the three subdomains are depicted by three different greens). (**d**) *Ft*PPDK in nucleotide-unbound state (PDB 5JVJ/A). (**e**) *Ft*PPDK in semi-closed, nucleotide-bound state (PDB 5JVL/C). (**f**) *Ft*PPDK in fully closed, nucleotide-bound state (PDB 5JVL/A).

**Figure 2:**
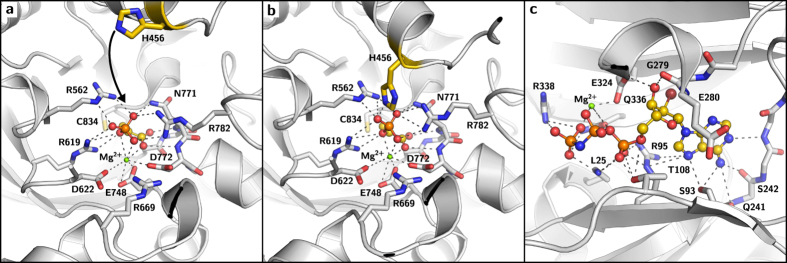
Substrate binding sites of *Ft*PPDK. (**a**) Semi-closed state of the PEP binding site (PDB 5JVL/A) with the catalytic H456 (yellow) pointing away from PEP. (**b**) Closed state of the PEP binding site (PDB 5JVL/C) revealing tight interactions between PEP and surrounding residues, including the catalytic H456 (yellow). (**c**) Closed state of the nucleotide binding site of 5JVL/D occupied with 2'-Br-dAppNHp. Interacting residues are highlighted.

The structure of the C_4_-isoform of PPDK from the flowering plant *Flaveria trinervia* was determined by molecular replacement at 2.9 Å resolution using the maize structure (PDB ID 1VBH)^11^ as a template. The structure (PDB 5JVJ) includes two monomers in the asymmetric unit (ASU) forming a dimer that corresponds to the previously described biological assembly of bacterial and maize PPDK^8^,^11^ with an overall well-defined electron density for the entire monomer A and for the PBD of monomer B. Parts of the NBD of monomer B revealed only poorly defined electron density, and direct tracing of monomer B in these regions was hampered. Yet, both monomers show electron density in the PBD for the co-crystallized substrate PEP. Besides, the NBD of monomer B exhibits additional density in both the *mF*_o_ − *DF*_*c*_ difference map and the feature enhanced maps (FEM, see methods section) probably reflecting a bound adenine nucleotide. The overall shape of this additional density is consistent with structural requirements and binding mode of adenine nucleotides in other nucleotide-binding proteins with the ATP-grasp fold^16^,^17^. In addition, this density complies with those observed when PPDK was crystallized in the presence of the nucleotide analogue 2′-Br-dAppNHp (see PDB 5JVL and Fig. 2c). However, since the molecular identity of the bound compound was not fully resolved at the present resolution, no compound was placed in this density in the deposited structure. Large parts of monomer B were successfully built using monomer A as a template by iterative manual model building and refinement. Yet, no conclusive electron density was found for residues 18–22, 47–65, 83–87, 101–106, 120–124, 163–166, 192–198 and 216–236. An overall root mean square deviation (RMSD) of 4.8 Å was calculated from a structural alignment of the individual monomers of the PPDK dimer in 5JVJ, indicating a substantial difference in their conformation. The main difference is found in the NBD of both monomers with the A monomer reflecting an open conformation and the B monomer reflecting a closed conformation of this domain. Overall, the orientation of NBD1 (aa 1–111 and 197–243) and NBD2 (aa 112–196) relative to NBD3 (aa 244–340) is greatly changed in the two monomer conformations (Fig. 1d–f). Superimposition of the NBD subdomains highlights that NBD1 is reoriented by a large motion of about 40° around a hinge region consisting of two short peptide linkers formed by residues 112–115 and 195–200 towards NBD3. At the same time, NBD2 is displaced by about 40° to accommodate for the new position of NBD1. The hinge motion of NBD1 and NBD2 results in the closing of the large cleft formed in the open configuration of the PPDK monomer (Fig. 1d–f). This cleft is no longer accessible in the closed form of the PPDK monomer. The presence of two structurally distinct conformations of the PPDK monomer (NBD open versus closed) within a single crystal structure may suggest that these structural asymmetries reflect functional asymmetries in substrate binding and/or catalytic turnover in the individual subunits of the PPDK dimer. A similar structural asymmetry in the monomer arrangement (open versus closed) was observed in the crystal structure of bacterial ATP-dependent DNA helicases and related to functional asymmetry in the mechanism of ATP hydrolysis in solution in each of the two subunits forming the functional dimer^12^,^18^.

When 2′-Br-dAppNHp was used for co-crystallization (PDB 5JVL), the crystal contains four monomers in the ASU arranged in terms of two dimers. The dimers correspond to the subunit arrangement in 5JVJ. Electron density maps (Supplementary Fig. 3) clearly indicate main chain connectivity and most of the side chain orientations for subunits A, C and D, whereas the majority of the NBD in chain B is not traceable. The overall structures of both *Ft*PPDK crystal forms (Fig. 1a,d,e,f) are highly similar to PPDKs from *C. symbiosum, T. brucei* and *Z. mays*^8^,^11^,^19^ consisting of an N-terminal NBD (aa 1–340), a CD (aa 380–515) of *β*-strands with associated *α*-helices that contain the catalytic H456 in helix 20 involved in the phosphoryl group transfer between NBD and PBD, and a C-terminal TIM barrel containing the PBD (aa 535–874). For monomers C and D of 5JVL, the catalytic H456 is located in close position and appropriate orientation to the bound PEP substrate to mediate phosphoryl group transfer from the NBD to the PBD (Fig. 2a,b). Such a close contact has never been observed in previously reported PPDK structures, but has been resolved in Enzyme I of the *E. coli* Phosphoenolpyruvate:Sugar Phosphotransferase System (PTS), a bacterial carbohydrate import system bearing a homologous PEP binding domain^20^. For structural comparison of the catalytic sites in PTS Enzyme I and the PPDK a set of reference atoms was selected. While the catalytic histidine in the PTS Enzyme I structure is in the phosphorylated state, the corresponding histidine in the active site of PPDK structure 5JVL is in the non-phosphorylated form. On the contrary, PPDK substrate PEP carries a phosphoryl group, whereas PTS Enzyme I substrate inhibitor oxalate does not. Hence, atoms *N*_*ε*2_ of the catalytic histidine oxygen atoms O2 in oxalate and PEP in 5JVL were used for distance measurements and structural comparison of both enzymes at their PEP binding site. In the PTS Enzyme I structure, the distance between the phosphorylated *N*_*ε*2_ atom of the catalytic H189 and the oxygen atom O2 of the oxalate substrate is 4.9 Å. The distance between *N*_*ε*2_ of the catalytic H456 and O2 of the PEP substrate in chain C and D of the 2'-Br-dAppNHp-*Ft*PPDK structure is similar (4.5 Å) (Fig. 2b), which emphasizes the close structural relationship of the newly described extreme conformation observed in 5JVL chains C and D and the PTS Enzyme I structure. This close spatial arrangement of the catalytic histidine and the phosphoryl group substrate in the PEP binding site enables efficient phosphoryl group transfer from PEP to H456 or H456 to pyruvate in PPDK as for PTS Enzyme I. The distance between the nucleophilic nitrogen atom *N*_*ε*2_ of H456 in the 2'-Br-dAppNHp-*Ft*PPDK structure and the attacked phosphorus of PEP is around 3 Å (Fig. 2b). This relatively short distance is indicative for an associative in-line displacement of the phosphoric monoester^21^,^22^. Therefore, the structural conformations resolved in the 2'-Br-dAppNHp-*Ft*PPDK structure visualize and verify for the first time the proposed phosphoryl group transfer mechanism in the PBD and the involvement of the catalytic histidine in the CD in this process.

Considering the overall CD, only small conformational changes between the plant structures from maize and *Flaveria* presented here and the non-plant structures from *Clostridium* and *Trypanosoma* are evident indicating that this domain primarily rotates as a rigid body. However, in contrast to the crystal structures of the non-plant PPDKs where the CD is close to the NBD, the CD of *Ft*PPDK rests alongside the PBD (Fig. 1a) as observed for PPDK from maize^11^ and the *Clostridium* triple mutant R219E/E271R/S262D (PDB 2R82)^9^. The different conformations of the NBD observed in the *Flaveria* structures presented in this work cover the whole range of conformational intermediates observed with other members of the ATP grasp family such as synapsin^23^ or biotin carboxylase^24^ and reflect individual snapshots of the closing motion of this domain. These structural intermediates of PPDK have been previously proposed based on structures of homologous nucleotide binding enzymes^25^ but have not been experimentally verified until now.

### Structural intermediate of the swiveling motion

**Figure 3:**
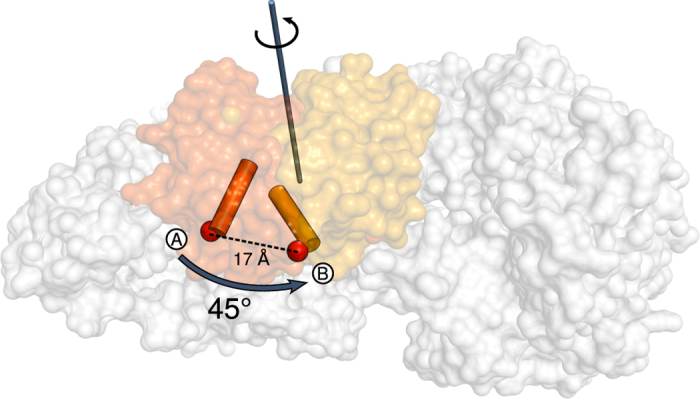
Stepped movement of the CD. CDs of 5JVL/C (A, orange) and 5JVN (B, yellow). Helix 20 containing the catalytic H456 is depicted as cylinder with the C_*α*_ atom of H456 shown as red sphere. The rotational axis for the transitions between states A and B is depicted as a blue arrow. The distance between the C_*α*_ atoms of the catalytic His456 is shown as a dashed line.

In contrast to the C_4_ plant crystal structures from *F. trinervia* and maize, the structure of the C_3_-isoform of PPDK from *F. pringlei* (Fig. 1b) contains only one monomer in the ASU (PDB 5JVN). Remarkably, in this structure the CD is positioned in an intermediate state between the NBD and PBD, thereby enlarging the solvent accessibility of the PEP binding pocket. The CD position is not enforced by crystal contacts and therefore represents a genuine structural intermediate of the proposed swiveling mechanism. The entire domain is rather mobile resulting in higher B-factors in this region. Consequently, the electron density of several side chains in this region is not well defined. Concerning the substrate and cofactor binding site, density is visible for both ligands, PEP and 2'-Br-dAppNHp. Additional density in the conformational intermediate was observed in close proximity of the phosphoryl group of the PEP substrate. This additional density might reflect an alternative position of the PEP substrate in the PBD. However, the available resolution and data quality prevent modelling of the PEP substrate in those positions in the active site. Compared to the near-PBD positioned conformation of the CD in the C_4_-isoform (PDB 5JVL/C and 5JVL/D) the entire CD in the *Fp*PPDK structure is rotated by 45° towards the NBD around a rotation axis formed by the linker region (Fig. 3). When compared to the PPDK structure from *Clostridium symbiosum* (*Cs*PPDK, PDB 1KBL)^10^, the CD is rotated in the opposite direction and displaced by 52° towards the PBD. Focusing on the catalytic H456 in the CD, this residue is displaced by 17 Å(C_*α*_-distance) when the *F. pringlei* conformational intermediate (5JVN) and the PBD-facing *Ft*PPDK structures (e.g. 5JVL/C) are aligned, and by 24 Å in the opposite direction when the NBD-facing conformation observed in the *Cs*PPDK is compared to the *Fp*PPDK structure, respectively (Supplementary Fig. 7). Hence, compared to the two extreme conformations of the CD next to the NBD or PBD previously resolved, the CD in the *F. pringlei* conformational intermediate shows about 50% of the rotational and translational movement of the proposed complete swiveling motion. The CD intermediate identified in our crystallographic studies suggests that the swiveling motion of this domain in the catalytic cycle proceeds via at least one sub-step, starting from a position near either the PBD (Fig. 1a) or the NBD (Fig. 1c) via the intermediate conformation resolved in 5JVN to a position near the other substrate binding site. Similarly, stepped movements have been described e.g. for the F_1_ ATPase rotary molecular motor^26^,^27^,^28^,^29^.

To elucidate critical contacts mediating interactions between the CD or linker domain (LD) and the PBD, the *Fp*PPDK conformational intermediate was analyzed with Cytoscape^30^ and RINalyzer^31^, which identified a salt bridge formed between E804 and R462, and non-polar patches at I864 in the PBD and L378 in the LD likely involved in hydrophobic interactions. Both interactions may restrict rotational freedom of the CD in this state, promoting the stepped motion of the CD. MD simulations confirm the presence of these interactions, although the salt bridge repeatedly forms and breaks (Supplementary Fig. 6f–h). Conservation analysis of these interactions by ConSurf^32^ revealed that only the non-polar patches are universally conserved among plant and non-plant PPDKs, whereas the identified salt bridge is unique to plant PPDKs, although non-plant enzymes might realize a corresponding interaction by an alternative salt bridge formed by R462 with an aspartate residue at position 801.

### Conformational space spanned by PPDK structures

**Figure 4:**
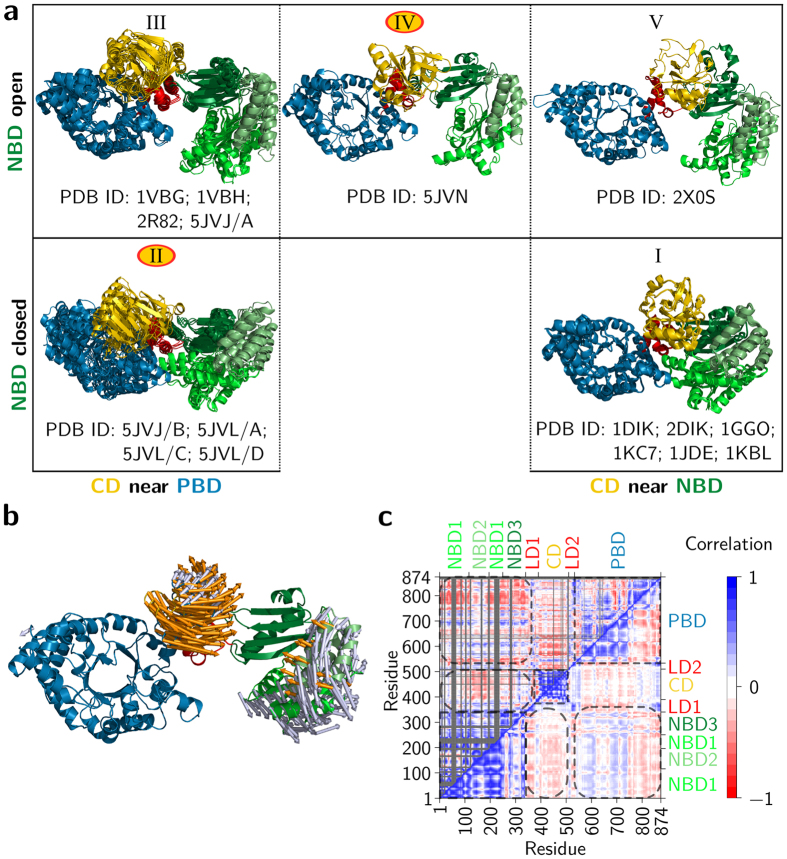
Motions of PPDK. (**a**) Overview of all currently known crystal structures of the PPDK clustered according to C_*α*_ atom RMSD (Roman numerals), showing the CD near the PBD (left), between PBD and NBD (middle), and near the NBD (right) as well as the NBD in an open (top) or closed (bottom) conformation. Conformational states observed for the first time in this work are marked with an orange circle. (**b**) Representation of atomic displacements along the directions of the first two principal components (1^st^: gold; 2^nd^: silver) obtained from a PCA over the representative structures of each cluster depicted in panel (**a**). The amplitudes of the motions were scaled, and a cutoff for small displacements was applied for best graphical representation. (**c**) Cross-correlation maps of C_*α*_ atom fluctuations of the representative structures of each cluster (upper triangular) and from an aggregate MD simulation time of ~10 μs (lower triangular). The two axes on the left and at the bottom refer to residue indices according to the *F. trinervia* numbering. Positive correlations are indicated in blue, negative correlations in red (see color scale). Residues not resolved in all compared crystal structures are shown in black. Substructures of PPDK are labeled on the top and right axes. Correlated movements within domains are marked with squares, correlated movements between the domains are marked with boxes with round corners.

To investigate the conformational space spanned by all available PPDK structures including the three newly resolved ones, the structures were clustered with respect to structural similarity. The clustering reveals five conformational states (Fig. 4a; Supplementary Tab. 2). The *Ft*PPDK structures described above constitute cluster II (PDB 5JVJ/B and 5JVL) and the *Fp*PPDK structure cluster IV (PDB 5JVN) (Supplementary Tab. 1 and Supplementary Tab. 2), demonstrating that these structures populate hitherto uncharted regions of PPDK’s conformational space. In addition, PDB 5JVJ/A supplements cluster III.

A principle component analysis in Cartesian coordinate space (PCA) across PPDK crystal structures corroborates the existence of two predominant motions: ~89% of the variance in the C_*α*_ atom coordinates can be explained by the first two principal components (PC) (Supplementary Fig. 4a). Atomic displacements along the component directions and a domain-wise index of the collectivity of motions (eq. 1, Supplementary Fig. 4b) reveal that the first PC predominantly characterizes a swiveling motion of the CD (Fig. 4b). The second PC characterizes a coordinated opening-closing motion of the NBD, with almost exclusively the first and second subdomain executing the movement (Fig. 4b and Supplementary Fig. 4b). Notably, the first PC also indicates a coupling of the swiveling motion of the CD with the opening-closing motion of the NBD as this PC leads to displacements in both domains (Fig. 4b). Cross-correlations of atomic fluctuations computed from the cluster-representative crystal structures agree very well with this result (Fig. 4c/upper triangle): They reveal, aside from positively correlated motions of the NBD, CD, and PBD themselves, weakly anti-correlated motions between the CD and the NBD (correlation coefficient down to −0.2), in agreement with the collective motions described by the first PC (Fig. 4b). Particularly, subdomains NBD1 and NBD2 move towards the CD if the latter swivels from the PBD to the NBD and vice versa.

To exclude any bias by the small number of available crystal structures, we further explored the conformational space of *Ft*PPDK by molecular dynamics (MD) simulations of in total ~10 μs length (Supplementary Tab. 3). Cross-correlations of atomic fluctuations computed from the MD simulations yield a qualitatively and quantitatively highly similar result compared with correlated motions computed from the cluster-representative crystal structures (Fig. 4c), confirming the anti-correlated motions between the CD and the NBD (correlation coefficient down to −0.15).

### Directionality of the swiveling motion

**Figure 5:**
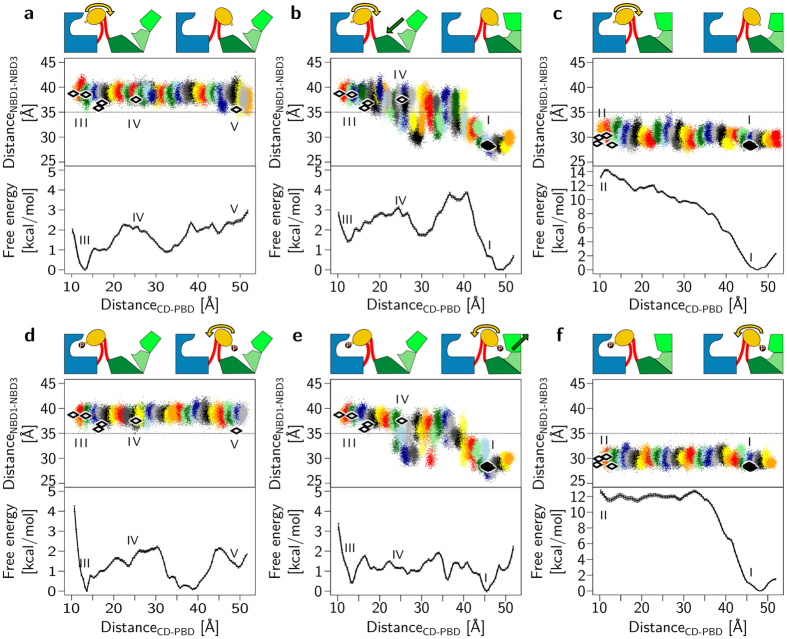
1D Potential of mean force of the swiveling motion of the CD with the distance H456_C*α*_ − H565_Cα_ used as a reaction coordinate. Results for the non-phosphorylated state are depicted in (**a–c**), those for the state with phosphorylated H456 in (**d–f**). (**a,d**) depict results obtained with open NBD, (**b,e**) those with a simultaneous opening-closing of the NBD, and (**c,f**) with closed NBD. At the top, schematic representations of each state at the respective endpoints of a PMF are shown. In the middle row, sampled conformations are projected onto distance_CD–PBD_ and distance_NBD1 − NBD3_ (Supplementary Fig. 4c), with each color representing one MD simulation with an umbrella potential applied at a given value of distance_CD–PBD_. The PMFs are depicted in the bottom row. The diamonds show projections of PPDK crystal structures in conformational states marked by Roman numbers (Fig. 4a) onto the plane spanned by the two reaction coordinates (using for each organism the corresponding residues to evaluate the reaction coordinates (Supplementary Tab. 1))

The above analyses suggest the *Fp*PPDK structure (cluster IV, Fig. 4a) to be an intermediate of the swiveling motion of the CD and indicate coupled motions between the CD and the NBD. To corroborate these findings, we computed the configurational free energy (potential of mean force, PMF) using the distance between H456_C*α*_–H565_C*α*_(distance_CD–PBD_, Supplementary Fig. 4c, numbering according to *F. trinervia* and *F. pringlei*, see also Supplementary Tab. 1) as a reaction coordinate. This reaction coordinate represents the swiveling motion very well (Supplementary Fig. 4d). PMFs were computed by umbrella sampling along distance_CD–PBD_ for three plausible transition paths obtained by targeted constrained geometric simulations^33^ between start/end states of *Ft*PPDK from cluster III/cluster V, cluster III/cluster I, and cluster II/cluster I (Fig. 4a and Supplementary Fig. 5a). These transition paths were chosen such that the swiveling motion occurs in the presence of an open NBD (Fig. 5a/d), a closed NBD (Fig. 5c/f), or where the NBD closes when the CD approaches it and vice versa (Fig. 5b/e). While the opening-closing motion of the NBD was not restrained during the umbrella sampling, the observed sampling very well represents these paths (Fig. 5). Distance_CD–PBD_ was sampled in intervals of 1 Å, leading to a sufficient overlap of the sampling windows (Fig. 5).

The obtained PMFs (Fig. 5a,b,d,e) show several remarkable characteristics. First, the overall precision is high, with standard deviations for all points <0.15 kcal mol^−1^. Second, the free energy difference between conformational states III and I, or III and V (Fig. 4) is <2 kcal mol^−1^ (Fig. 5a,b,d,e), showing that the respective higher-energy conformational state is populated to ~2% at room temperature. Third, we identify a stable conformational intermediate of the CD swivelling motion, revealed by pronounced minima in the PMFs at a distance_CD–PBD_ ~30–35 Å (Fig. 5a,b,d,e), which is structurally highly similar to the intermediate state IV found in the *Fp*PPDK crystal structure (located at a distance_CD–PBD_ ~25 Å) as shown by a C_*α*_ atom RMSD of 2.7 Å. Note that the *Fp*PPDK crystal structure was not used for generating the transition path, hence, no information about conformational state IV entered the PMF calculations. The presence of the intermediate state leads to a sawtooth-like PMF. Fourth, the PMFs reveal for PPDK with non-phosphorylated H456 (Fig. 5a,b) that the correlated movement of the CD and the NBD results in state I being favored over III by 1.4 kcal mol^−1^ (Fig. 5b); in contrast, if the CD moves with the NBD remaining open, state V is disfavored over III by 2.1 kcal mol^−1^ (Fig. 5a). Therefore, movement of the non-phosphorylated CD from the PBD towards the NBD is exergonic only if it is coupled to a closing motion of the NBD, which is in line with the swiveling domain model^8^. This finding corroborates cross-correlations between these motions observed from crystal structures (Fig. 4c, upper triangle) and from structures obtained by MD simulations (Fig. 4c, lower triangle). Fifth, the PMFs suggest that the phosphorylation state of the CD influences the preferred direction of motion of this domain: When H456 is phosphorylated, state III becomes favorable over V by 1.5 kcal mol^−1^ (Fig. 5d) or is only slightly disfavored over I by 0.5 kcal mol^−1^ (Fig. 5e). Compared to the non-phosphorylated state, the sawtooth-like PMF is thus tilted towards III. Therefore, with phosphorylated H456, the movement of the CD from the NBD towards the PBD is exergonic or approximately isoenergetic. This result is also in line with the swiveling domain model^8^. In the phosphorylated state, coupled motions between the CD and the NBD have a smaller influence on the energetics of the conformational states. We speculate that the electrostatic repulsion between the phosphate group at H456 and reaction products, including adenosine monophosphate, still bound to or being in the vicinity of the NBD, fosters the CD movement towards the PBD instead.

### Complex, stepped swiveling motion

**Figure 6:**
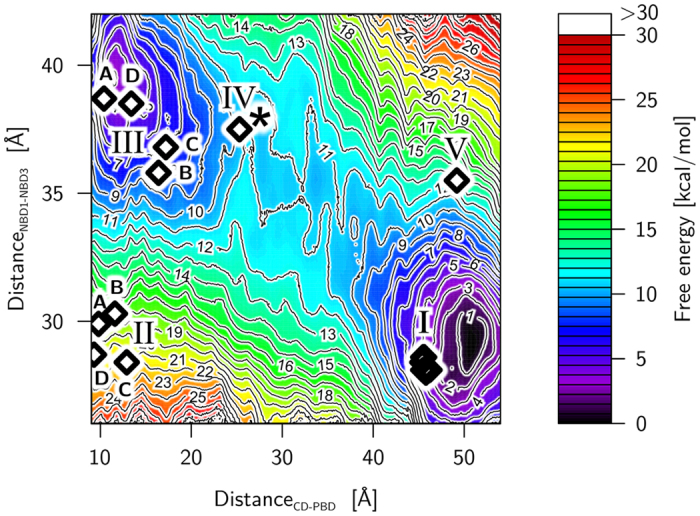
2D Potential of mean force, with the distance_CD–PBD_ between H456_C*α*_ – H565_C*α*_ and the distance_NBD1−NBD3_ between S215_C*α*_ − E272_C*α*_ used as reaction coordinate of the swiveling motion of the CD and the opening-closing motion of the NBD, respectively, (Supplementary Fig. 4c) computed for H456 in the non-phosphorylated state. The diamonds show projections of PPDK crystal structures in conformational states marked by Roman numbers (Fig. 4a) onto the plane spanned by the two reaction coordinates (using for each organism the corresponding residues to evaluate the reaction coordinates (Supplementary Tab. 1)) (I: PDB 1DIK, 2DIK, 1GGO, 1JDE, 1KBL, 1KC7; II_A_: 5JVJ/B, II_B_: 5JVL/A, II_C_: 5JVL/C, II_D_: 5JVL/D; III_A_: 2R82, III_B_: 1VBG, III_C_: 1VBH, III_D_: 5JVJ/A; IV: 5JVN; V 2X0S). The star marks a shallow energy minimum close to conformation IV.

PMFs calculated for the transition between conformational states I and II unexpectedly do not reveal a structural intermediate along the transition path and show a marked prevalence of state I (free energy difference >12 kcal mol^−1^), irrespective of the phosphorylation state of H456 (Fig. 5c,f). To provide an explanation for this observation and further details on the coupling between motions of the CD and the NBD, we computed a 2D PMF, using as reaction coordinates distance_CD–PBD_ and the distance between *S*215_C*α*_–E272_Cα_ (distance_NBD1−NBD3_, Supplementary Fig. 4c, numbering according to *F. trinervia* and *F. pringlei*, see also Supplementary Tab. 1) for the non-phosphorylated PPDK. The reaction coordinate distance_NBD1−NBD3_ represents the opening-closing motion of the NBD very well (Supplementary Fig. 4e). Reference points for umbrella sampling were generated using targeted constrained geometric simulations^33^ between start/end states of *Ft*PPDK from cluster III/cluster I (Supplementary Fig. 5a), with further reference points added using the conformation after 6 ns of umbrella sampling as a starting point for the next interval. The sampling windows overlap well along the two reaction coordinates (Supplementary Fig. 5b–d), and the 2D PMFs are qualitatively indistinguishable irrespective whether only the first half, the second half, or the complete sampling time is used for their calculation (Supplementary Fig. 5e-g), strongly indicating converged results. The most prominent feature of the PMF is that conformational states III and I reside in or close to minima of the free energy landscape (Fig. 6). Furthermore, a shallow free energy minimum is identified at distance_CD–PBD_ ∼28 Å and distance_NBD1−NBD3_ ∼38 Å (marked by a star in Fig. 6), lying close to the structural intermediate from the *Fp*PPDK crystal structure (conformational state IV, Fig. 4a). The transition path between conformational state III and IV runs such that distance_NBD1−NBD3_ remains almost constant while distance_CD–PBD_ changes by ~15 Å. In contrast, between conformational state IV and I, the transition paths continues in a diagonal manner, revealing a correlation between the swiveling motion of the CD (change of distance_CD–PBD_ by ~25 Å) and the opening-closing motion of the NBD (change of distance_NBD1−NBD3_ by ~10 Å). These characteristics agree very well with those found by the 1D PMFs, where distance_NBD1−NBD3_ only starts to decrease once distance_CD–PBD_ >25 Å (Fig. 5b/e). Overall, both PMF calculations thus reveal a complex, stepped swiveling motion of the CD with varying degrees of coupling to the NBD motions and proceeding via conformational state IV. Moreover, the 2D PMF reveals that conformational state II is located in a region of elevated free energy (Fig. 6), providing an explanation why a transition path obtained by targeted simulations between conformational states II and I results in a downhill motion towards state I (Fig. 5c,f). Unrestrained MD simulations confirm this finding (see section Supporting Results in the SI, Supplementary Fig. 6). A plausible explanation for why this state is observed in PDB 5JVL may be that protein-protein interactions between the PBD of chain B and the CD of chain A occur as crystal contacts, which can stabilize the CD near the PBD despite a closed NBD.

### Effective driving force of the swiveling motion

The analysis of crystal structures, unrestrained MD simulations, and configurational free energy calculations consistently and independently revealed the existence of structural intermediates of the swiveling motions and coupled motions of the CD and the NBD for non-phosphorylated PPDK. The respective 1D PMF of PPDK with non-phosphorylated H456 is sawtooth-like (Fig. 5b), with barrier heights for the transition from state III to I between 1.5–2.5 kcal mol^−1^ (2.5–4.1 *kT* at *T* = 300 K). For the reverse transition with phosphorylated H456, the 1D PMF also displays a sawtooth-like character (Fig. 5e), with barrier heights of 1.8 kcal mol^−1^ (3 *kT* at *T* = 300 K) and the free energy profile being tilted in favor of state III compared to the non-phosphorylated PPDK. The sawtooth-like free energy profiles suggest that PPDK can exploit random thermal fluctuations for directional motion of its CD. Typically, this type of profile is indicative of a Brownian ratchet mechanism^34^, pioneered by Feynman^35^ and Huxley^36^. Barrier heights on the order of *kT*, as associated with the CD motion in the PPDK catalytic cycle, suggest that a Brownian ratchet biases fluctuations rather than rectifying them^37^. To drive the respective directional motions, a non-equilibrium situation needs to be created that relaxes towards equilibrium. For the non-phosphorylated PPDK, this situation is suggested to be created by binding of ATP to the NBD, as such binding leads to a closing of the NBD due to the progressive formation of ATP/NBD interactions. Transmitted via coupled motions, the NBD closing then leads to a preference for the CD to be close to the NBD, at least in the second half of the transition pathway between PBD and NBD. However, we are aware that biased Brownian ratchets and power stroke motors have the same phenomenological behavior and are difficult to distinguish experimentally^37^. Hence, we cannot exclude that the conformational changes observed in the NBD of our structural intermediates induce a strain in the enzyme that would directly drive the CD motion upon ATP binding, thus resulting in a power stroke mechanism^38^. Overall, this situation is similar to ATP synthase and ATP-dependent molecular machines such as myosin, kinesin, or chaperonin parts, where the stepped motions in the catalytic cycle are triggered by binding of the high energy substrates PEP or ATP, respectively^27^. For the phosphorylated PPDK, at least part of the non-equilibrium situation is suggested to arise from electrostatic repulsion between the phosphorylated H456 and the NBD. A similar situation was created by introducing appropriately charged residues into a mutant of non-phosphorylated *Cs*PPDK, resulting in the CD being adjacent near the PBD (PDB ID 2R82)^9^. In all, our analyses suggest that both changes of the binding state of PPDK and of its molecular identity ((non)phosphorylation) contribute to the enzyme acting as a molecular switch with respect to the swiveling motion.

Moreover, our crystallographic and molecular simulation data are indicative that PPDK might employ a Brownian ratchet mechanism biasing thermal fluctuations in order to generate a net directional CD motion. The coupling of this motion to the open-close state of the NBD revealed by the distinct conformational states (Fig. 1a–c and Supplementary Fig. 5a) and substrate binding states resolved in our dimeric structural intermediate of the PPDK catalytic cycle further suggests that the enzyme might employ an alternate binding change mechanism similar to ATP synthase or bacterial ATP-dependent DNA helicases.

## Methods

### Expression and purification of recombinant *Ft*PPDK/*Fp*PPDK

Codon-optimized coding regions of PPDK from *Flaveria trinervia* (EMBL-ENA: X57141)^39^ or *Flaveria pringlei* (EMBL-ENA: X75516)^40^, stripped of the chloroplast transport sequence, were cloned into the multiple cloning site of the pET-16b vector (Novagen) including a histidine_10_ tag and a Tobacco Etch Virus (TEV) protease cleavage site. The plasmid was used to transform *E. coli* BL21 (DE3) cells (Agilent Technologies). Previously described expression and purification protocols^11^ were adapted for *Ft*PPDK/*Fp*PPDK. Transformed *E. coli* BL21 (DE3) cells (Agilent Technologies) were grown in 2YT medium (5 gL^−1^ NaCl, 10 gL^−1^ yeast extract, 16 gL^−1^ peptone) with ampicillin at 30 °C to OD_600_ = 0.8. Protein expression was induced by the addition of 0.1 mM isopropyl-*β*-D-thiogalactopyranosid (IPTG). Cells were harvested 18 after induction by centrifugation. After harvesting, cells were suspended in lysis buffer (50 mM Tris/HCl pH 7.5, 300 mM NaCl, 10 mM imidazole, 10 mM MgSO_4_, 10% (w/v) glycerol, 5 mM DTT, 0.002% (w/v) phenylmethanesulfonylfluoride) and disrupted using a cell disruptor (Constant Systems). PPDK was purified using a nickel affinity chromatography column (GE Healthcare) using purification (50 mM Tris/HCl pH 7.5, 300 mM NaCl, 10 mM MgSO_4_, 10% (w/v) glycerol, 5 DTT) and elution buffer (50 mM Tris/HCl pH 7.5, 300 mM NaCl, 500 mM imidazole, 10 mM MgSO_4_, 10% (w/v) glycerol, 5 mM DTT). The loaded column was washed with 50 mM and 150 mM imidazole, 500 mM imidazole were used for the elution of PPDK. A PD-10 desalting column (GE Healthcare) was used to exchange the elution buffer against purification buffer before enzymatic cleavage of the affinity tag via Tobacco Etch Virus Protease (TEV) over-night at room temperature. Cleaved PPDK was concentrated by ultrafiltration (30 kDA cutoff, Millipore) and the buffer was exchanged using a PD-10 column. For crystallization trials, crystallization buffer (10 mM Tris/HCl pH 7.5, 5 mM MgSO_4_) was used for the buffer exchange step, otherwise PPDK storage buffer (50 mM Tris/HCl pH 8, 10 mM MgCl_2_, 0.1 mM EDTA, 5 mM DTT). Monodispersity of the sample was verified via size exclusion chromatography (SEC) and dynamic light scattering (DLS) (Supplementary Fig. 2a). Activity of purified PPDKs was confirmed by a coupled-enzyme assay in the PEP-forming direction^41^.

### Crystallization

Initial crystallization trials were performed in microbatch technique. *Ft*PPDK in crystallization buffer at a concentration of 10 mg mL^−1^ was incubated at room temperature for 10 min with 20 mM phosphoenolpyruvate (PEP) and either 10 mM nicotinamide adenine dinucleotide (NADH) or 1.5 mM 2'-Br-dAppNHp. The protein solution was mixed with precipitant at a 1:1 ratio resulting in a final volume of 2 μL. The drop was sealed using mineral oil (Sigma Aldrich). The optimized composition of the precipitant solution included 17% (w/v) PEG 3350, 100 mM MOPS (pH 7) and 100 mM magnesium formate with either 10 mM nicotinamide adenine dinucleotide (NADH) or 1.5 mM 2'-Br-dAppNHp added to the protein solution 10 min prior of mixing with the precipitant. Crystals sized approx. 500 × 40 × 10 μm^3^ appeared within 24 h after incubation at 21 °C, were transferred into a cryoprotection buffer comprised of the precipitant solution supplemented with 20% ethylene glycol and cryo-cooled in liquid nitrogen.

*Fp*PPDK was crystallized in sitting drop technique. *Fp*PPDK in crystallization buffer at a concentration of 10 mg mL^−1^ was incubated for 10 min with 20 mM PEP and 2 mM 2'-Br-dAppNHp at room temperature. The optimized reservoir solution was composed of 85 mM HEPES pH 7.5, 17% (w/v) PEG 4000, 15% (w/v) glycerol and 5% (v/v) isopropanol and mixed at a 1:1 ratio with the protein solution in a final drop volume of 2 μL. Crystals sized approx. 200 × 50 × 50 μm^3^ appeared within 48 h after incubation at 12 °C. The crystals were flash-frozen in liquid nitrogen without adding any additional cryoprotectant.

### Structure determination

X-ray diffraction data for *Ft*PPDK were collected at the European Synchrotron Radiation Facility (ESRF), Grenoble at beamline BM30A at 0.9799 Å wavelength for 5JVJ with an oscillation range of 1° per image spanning a total range of 151° at 100 K. Data for the 2'-Br-dAppNHp-bound form was collected at 0.9123 Å with an oscillation range of 0.5° per image and 360° of total range. The data were integrated and scaled with XDS^42^ and data reduction was performed with Aimless^43^ from the CCP4 suite^44^. Initial phases for 5JVJ were obtained by molecular replacement (MR) with *Z. mays* PPDK^11^ as a template (PDB 1VBH, 79% sequence identity) using Phaser^45^. The initial model included two monomers in the asymmetric unit (ASU). For one monomer (chain B) only the PBD was correctly placed in the electron density, hence the misplaced CD and NBD were removed manually. This model was subjected to multiple rounds of manual model rebuilding and extension using Coot^46^ and refinement by phenix.refine^47^ using local non-crystallographic symmetry restraints to account for the obvious conformational differences between both monomers. Parts of chain B were gradually retrieved in this process. The structure of the 2'-Br-dAppNHp-bound *Ft*PPDK (5JVL) was determined by MR using the coordinates of the previously determined 5JVJ/A structure. The resulting model contained four monomers in the ASU. Three monomers were extended to completeness, the fourth monomer (chain B) exhibited inconclusive density for the NBD. The atomic displacement parameters (ADPs) for both crystal forms were refined individually and were partly described as groups of translation, libration and screw-motion (TLS)^48^.

Data of the *Fp*PPDK were collected at the ESRF beamline ID29 at a wavelength of 0.9762 Å with an oscillation range of 0.1° per image and 360° of total range. The reflection data were processed with XDS. Initial phases were obtained by MR with Phaser using the PBD and NBD of 5JVL/D as starting model. The resulting structure was subject to automated model-rebuilding using Bucaneer^49^ which recovered the CD followed by iterative rounds of manual model rebuilding using Coot and refinement with phenix.refine and REFMAC5^50^. In all cases, ligands were not modeled into excess density indicated by the *mF*_o_ − *DF*_*c*_ map (where *m* represents the figure of merit, *D* the *σ*-A weighting factor and *F*_*o*_ and *F*_*c*_ the observed (experimental) and calculated (model) amplitudes respectively) until near-final refinement rounds to reduce model bias. To enhance sensitivity for weak sidechain features, Feature Enhanced Maps (FEM) were used in the model building process. For FEM, a 2*mF*_*o*_ − *DF*_*c*_ *σ*-A-weighted map is modified to strengthen weak signals if present. This includes calculation of omit maps, map randomization and map sharpening. The resulting map shows enhanced sensitivity for weak features and reduced model bias compared to 2*mF*_*o*_ − *DF*_*c*_ maps^51^. The structure was validated using tools provided by Coot and PHENIX, in particular MolProbity^52^. Figures were generated using PyMOL^53^. Final parameter of refinement and model stereochemistry of *Flaveria* PPDK structures 5JVJ, 5JVL, and 5JVN are summarized in Table 1.

### Comparison of PPDK crystal structures

All currently available crystal structures of PPDK were obtained from the Protein Data Bank^54^ (PDB) (Supplementary Tab. 1). The structure of PDB 5JVL, chain B and NMR derived structure of the CD only (PDB 2FM4) was excluded from further analysis as the NBD has not been resolved there. A multiple sequence and structural alignment of the PPDK structures was generated with PROMALS3D^55^. Residues resolved and common to all structures were identified from the alignment and used for further analysis.

### Cluster analysis of PPDK conformations

Cluster analysis on all PPDK crystal structures listed in Supplementary Tab. 1 was performed with the CPPTRAJ^56^ module of the AMBER suite of programs^57^ using the hierarchical agglomerative (bottom-up) algorithm. As a distance measure, the best-fit C_*α*_ atom root mean square deviation (RMSD) of all residues common in the PPDK structures with all domains resolved (Supplementary Tab. 1) was used. A maximal distance between all members of two clusters (complete linkage) of <4 Å was used as terminating criterion for the clustering. For subsequent analysis, only those crystal structures were used that are cluster representatives in order to avoid a bias towards the number of crystal structures in the same conformational state.

### Principle component analysis

To describe the essential dynamics of PPDK, a principle component analysis (PCA) in Cartesian space was performed on a set of experimental structures (Supplementary Tab. 1) and snapshots of MD simulations (Supplementary Tab. 3) using CPPTRAJ^56^ in a similar manner as described in refs 58 and 59. In detail, the coordinate covariance matrix was calculated for all C_*α*_ atoms present in all crystal structures without missing domains. The structures were RMS-fit to the average structure of all cluster representative crystal structures using the 15% least fluctuating residues to remove global transitional and rotational motion prior to calculating the coordinate covariance matrix. The symmetric matrix is diagonalized by an orthogonal coordinate transformation, yielding the eigenvalues and eigenvectors (principle components). An eigenvalue corresponds to the mean square eigenvector coordinate fluctuation (the variance) and, hence, describes how much a principal component contributes to the total coordinate fluctuations^60^.

To analyze the locality or collectivity of motions for the domains of PPDK, the collectivity index *κ* described in ref. 61 was calculated (eq. 1)


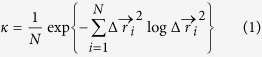


with *N* being the number of atoms in the domain, and 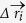 being the relative displacement of the principal component. All values of 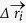 were scaled consistently such that 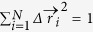. A value of *κ* = 1 indicates a mode of maximal collectivity, that is, all 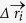 are identical. Conversely, if only one atom is affected by the mode, *κ* reaches the minimal value of 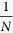.

### Homology modeling of *Ft*PPDK conformational states

As there was no crystal structure of *Ft*PPDK available at the beginning of this project, homology models of conformational states I, III, IV, and V were generated. From each cluster (Fig. 4a), the crystal structures with the highest resolution and without mutations that interfere with the enzymatic activity were used as templates (*C. symbiosum* structure 1KBL and 1KC7 for state I; *Z. mays* structure 1VBH and 1VBG for state II; *F. pringlei* structure 5JVN for state IV; and *T. brucei* structure 2X0S for state V). The sequence alignment of the *Ft*PPDK sequence and the respective template sequence(s) was generated by MAFFT^62^. The sequence identities are for conformational state I: 55%; conformational state III: 79%; conformational state IV: 96%; conformational state V: 54%. The homology models were generated using the program MODELLER^63^ in a multi-template approach, applying the dope loop model algorithm, and including ligands (if present in the crystal structure). The quality of our models was assessed for conformational state III, for which now PDB ID 5JVJ, chain A is available: 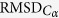 for NBD: 1.16 Å, for CD including the linker domain: 1.13 Å, and for the PBD: 0.93 Å. Such structural deviations are close to the experimental uncertainty of the crystal structure.

### Molecular dynamics simulations of *Ft*PPDK

The crystal structure of *Ft*PPDK (5JVL/C; conformational state II) and homology models of *Ft*PPDK for conformational states I, III, IV and V served as input structures for MD simulations. Three independent replicates of MD simulations were performed for each system which are summarized in Supplementary Tab. 3. Co-crystallized water and ligands were removed. Hydrogen atoms were added using REDUCE^64^, flipping side chains of Asn, Gln, and His when appropriate. These model systems were placed in a truncated octahedral box of TIP3P water^65^ leaving a distance of at least 11 Å between the solute and the border of the box. Counter ions were added to neutralize the systems. All MD simulations were performed with the ff99SB force field^66^ using the Amber suite of programs^57^. Parameters for the phosphorylated histidine were obtained from ref. 67. Bonds containing hydrogen atoms were constrained using the SHAKE algorithm^68^, and long range interactions were treated by the particle mesh Ewald (PME) method^69^. A time step of 2 fs was used. The system was equilibrated by, first, applying harmonic restraints to solute atom positions with force constants of at least 5 kcal mol^−1^ Å^−2^ for 100 steps of steepest descent and 400 steps of conjugate gradient minimization. Second, the temperature of the system was raised from 100 K to 300 K in 50 ps of NVT-MD simulations. Third, 150 ps of NPT-MD simulations were performed to adjust the system density. Finally, the force constants of harmonic restraints were gradually reduced to zero during 250 ps of NVT-MD simulations. Production NVT-MD simulations were carried out at 300 K, using the Berendsen thermostat^70^ and a coupling constant of 0.5 ps. Three independent replicates of MD simulations were performed for each system by spawning production runs after the thermalization at temperatures of 299.9 K, 300.0 K, and 300.1 K respectively. The first 2 ns of each trajectory were omitted from subsequent analyses. All unrestrained MD simulations are listed in Supplementary Tab. 3.

### Generation of transition paths

For the potential of mean force calculations, plausible pathways of the swiveling motion and opening-closing motion have been generated using targeted normal mode-based geometric simulations by the NMSim approach^33^. NMSim is a three-step protocol for multiscale modeling of protein conformational changes that incorporates information about preferred directions of protein motions into a geometric simulation algorithm. In the first step, the protein structure is coarse-grained by the software FIRST^71^ into rigid parts connected by flexible links. For this, an energy cut-off for including hydrogen bonds (and salt bridges) of −1 kcal mol^−1^ and a distance cutoff for including hydrophobic constraints of 0.35 Å were used. In the second step, low-frequency normal modes are computed by rigid cluster normal mode analysis (RCNMA) with a 10 Å distance cutoff for considering interactions between C_*α*_-atoms. In the third step, a linear combination of the first 50 normal modes was used to bias backbone motions along the low-frequency normal modes, while the side chain motions were biased towards favored rotamer states, generating 500 conformations in 500 simulation cycles with a step size of 0.5 Å and side chain distortion of 0.3. Targeted NMSim calculations^33^ were performed between start / end states of cluster III / cluster V, cluster III / cluster I, and cluster II / cluster I, using homology models for cluster III, V, and I, and the cluster-representative crystal structure for cluster II.

### Potential of mean force calculations

Free energy profiles of the swiveling motion of the CD (and the opening-closing motion of the NBD) were computed along the NMSim-generated transition paths by umbrella sampling^72^ followed by the Weighted Histogram Analysis Method (WHAM)^73^. As a reaction coordinate for analyzing *Ft*PPDK’s swiveling motion, the distance_CD–PBD_ between H456_C*α*_ – H565_C*α*_ was used (Supplementary Fig. 4c), as it changes monotonously between the two endpoints and provides an intuitive measure for the progress of the swiveling movement of the CD. The opening-closing motion of the NBD of *Ft*PPDK was analyzed along the reaction coordinate distance_NBD1−NBD3_, measured between S215_C*α*_–E272_C*α*_ (Supplementary Fig. 4c). 1D (2D) umbrella sampling MD simulations were performed along reaction coordinate(s) distance_CD–PBD_ between 10 Å and 52 Å (9 Å and 54 Å) (and distance_NBD1−NBD3_ between 26 Å and 42 Å) in intervals of 1 Å, applying harmonic potentials with a force constant of 1 kcal mol^−1^ Å^−2^ to tether the conformations to the respective reference point. This resulted in 42 (782) umbrella sampling simulations, each 42.5 ns (9 ns) long, excluding the first 2.5 ns (1 ns) from the WHAM analysis, for the 1D (2D) PMF. Approximately Gaussian-shaped frequency distributions were obtained for each reference point along the reaction coordinate(s), with all such distributions well overlapping (Supplementary Fig. 5b-d). The latter is a prerequisite for the successful application of WHAM^73^ to extract a PMF from these distributions. Bootstrapping was applied to compute the standard deviations at the reference points.

## Additional Information

**Accession codes:** Coordinates of dimeric and 2'-Br-dAppNHp-bound FtPPDK have been deposited in the Protein Data Bank under accession codes 5JVJ and 5JVL. Coordinates of *FpPPDK* have been deposited under the accession code 5JVN.

**How to cite this article**: Minges, A. *et al*. Structural intermediates and directionality of the swiveling motion of Pyruvate Phosphate Dikinase. *Sci. Rep.* **7**, 45389; doi: 10.1038/srep45389 (2017).

**Publisher's note:** Springer Nature remains neutral with regard to jurisdictional claims in published maps and institutional affiliations.

## Supplementary Material

Supplementary Information

## Figures and Tables

**Table 1:** Data collection and refinement statistics.

	5JVJ	5JVL	5JVN
**Data collection**			
Wavelength (Å)	0.9799	0.9193	0.9763
Space group	C121	P1	P622
Cell dimensions			
a, b, c (Å)	208.74, 69.04, 166.76	69.98, 108.44, 152.75	249.34, 249.34, 84.01
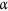 , 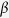 , 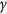 (°)	90, 112.84, 90	106.22, 101.81, 98.32	90, 90, 120
Resolution range (Å)	30.74–2.898 (3.002–2.898)	19.79–2.90 (3.003–2.90)	49.54–2.90 (3.040-2.-90)
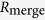	0.1013 (0.4002)	0.0965 (0.4446)	0.089 (0.597)
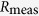	0.1231 (0.4847)	0.1119 (0.5155)	0.09513 (0.6552)
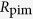	0.0689 (0.2696)	0.05663 (0.2604)	0.03553 (0.2468)
I/ 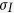	11.18 (3.13)	13.63 (3.21)	13.9 (2.7)
Wilson B (Å^2^)	39.69	39.21	79.98
Completeness (%)	98 (98)	98 (99)	100 (100)
Multiplicity	3.2 (3.2)	3.9 (3.9)	6.9 (6.8)
**Model and refinement**			
Reflections (unique/test)	48150/1227	90103/1874	34536/1695
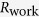 / 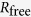 (Å)	23.7/26.1	21.2/23.2	20.0/23.5
No. of atoms			
Protein	11928	23511	6518
Ligand/ion	22	140	42
B-factors			
Protein	59.21	46.99	90.41
Ligands	29.62	28.59	116.36
RMSD			
Bonds lengths (Å)	0.002	0.017	0.010
Bond angles (°)	0.46	1.45	1.11
Ramachandran analysis			
Favored regions (%)	96.8	98.3	96.7
Allowed regions (%)	3.1	1.7	3.3
Outliers (%)	0.1	0.0	0.0

Highest resolution shell is shown in parentheses.

## References

[b1] Edwards G. E., Nakamoto H., Burnell J. N. & Hatch M. D. Pyruvate, Pi Dikinase and NADP-Malate Dehydrogenase in C4 Photosynthesis: Properties and Mechanism of Light/Dark Regulation. Ann. Rev. Plant Physio. 36, 255–286 (1985).

[b2] Chastain C. J. . Functional evolution of C4 pyruvate, orthophosphate dikinase. J. Exp. Bot. 62, 3083–3091 (2011). 21414960 10.1093/jxb/err058

[b3] Hatch M. D. Regulation of C 4 Photosynthesis: Factors Affecting Cold-Mediated Inactivation and Reactivation of Pyruvate, P I Dikinase. Aust. J. Plant Physiol. 6, 607 (1979).

[b4] Shirahashi K., Hayakawa S. & Sugiyama T. Cold Lability of Pyruvate, Orthophosphate Dikinase in the Maize Leaf. Plant Physiol. 62, 826–830 (1978). 16660615 10.1104/pp.62.5.826 PMC1092230

[b5] Sugiyama T. Purification, molecular, and catalytic properties of pyruvate phosphate dikinase from the maize leaf. Biochemistry-US. 12, 2862–2868 (1973). 10.1021/bi00739a014 4719122

[b6] Chastain C. J. & Chollet R. Regulation of pyruvate, orthophosphate dikinase by ADP-/Pi-dependent reversible phosphorylation in C3 and C4 plants. Plant Physiol. Bioch. 41, 523–532 (2003).

[b7] Chastain C. J., Heck J. W., Colquhoun T. A., Voge D. G. & Gu X.-Y. Posttranslational regulation of pyruvate, orthophosphate dikinase in developing rice (*Oryza sativa*) seeds. Planta 224, 924–934 (2006). 16596412 10.1007/s00425-006-0259-3

[b8] Herzberg O. . Swiveling-domain mechanism for enzymatic phosphotransfer between remote reaction sites. P. Natl. Acad. Sci. USA. 93, 2652–2657 (1996). 10.1073/pnas.93.7.2652 PMC39685 8610096

[b9] Lim K. . Swiveling Domain Mechanism in Pyruvate Phosphate Dikinase. Biochemistry-US. 46, 14845–14853 (2007). 10.1021/bi701848w 18052212

[b10] Herzberg O. . Pyruvate site of pyruvate phosphate dikinase: crystal structure of the enzyme-phosphonopyruvate complex, and mutant analysis. Biochemistry-US. 41, 780–787 (2002). 10.1021/bi011799+ 11790099

[b11] Nakanishi T., Nakatsu T., Matsuoka M., Sakata K. & Kato H. Crystal Structures of Pyruvate Phosphate Dikinase from Maize Revealed an Alternative Conformation in the Swiveling-Domain Motion. Biochemistry-US. 44, 1136–1144 (2005). 10.1021/bi0484522 15667207

[b12] Korolev S., Hsieh J., Gauss G. H., Lohman T. M. & Waksman G. Major Domain Swiveling Revealed by the Crystal Structures of Complexes of *E. coli* Rep Helicase Bound to Single-Stranded DNA and ADP. Cell 90, 635–647 (1997). 9288744 10.1016/s0092-8674(00)80525-5

[b13] Nguyen K. & Whitford P. C. Steric interactions lead to collective tilting motion in the ribosome during mRNA–tRNA translocation. Nat Comms 7, 10586 (2016). 10.1038/ncomms10586 PMC4742886 26838673

[b14] Schuwirth B. S. Structures of the Bacterial Ribosome at 3.5 A Resolution. Science 310, 827–834 (2005). 16272117 10.1126/science.1117230

[b15] Zhang Z. . Electron transfer by domain movement in cytochrome bc1. Nature 392, 677–684 (1998). 9565029 10.1038/33612

[b16] Qi X. . Structural basis of rifampin inactivation by rifampin phosphotransferase. P. Natl. Acad. Sci. USA 113, 3803–3808 (2016). 10.1073/pnas.1523614113 PMC4833264 27001859

[b17] Weiße R. H.-J., Faust A., Schmidt M., Schönheit P. & Scheidig A. J. Structure of NDP-forming Acetyl-CoA synthetase ACD1 reveals a large rearrangement for phosphoryl transfer. P. Natl. Acad. Sci. USA. 113, 519–528 (2016). 10.1073/pnas.1518614113 PMC4747732 26787904

[b18] Wong I. & Lohman T. M. A two-site mechanism for ATP hydrolysis by the asymmetric rep dimer p2s as revealed by site-specific inhibition with ADP-AlF4. Biochemistry 36, 3115–3125 (1997). 9115987 10.1021/bi9621977

[b19] Cosenza L. W., Bringaud F., Baltz T. & Vellieux F. M. The 3.0Å Resolution Crystal Structure of Glycosomal Pyruvate Phosphate Dikinase from *Trypanosoma brucei*. J. Mol. Biol. 318, 1417–1432 (2002). 12083528 10.1016/s0022-2836(02)00113-4

[b20] Teplyakov A. . Structure of phosphorylated enzyme I, the phosphoenolpyruvate:sugar phosphotransferase system sugar translocation signal protein. P. Natl. Acad. Sci. USA. 103, 16218–16223 (2006). 10.1073/pnas.0607587103 PMC1618308 17053069

[b21] Mildvan A. S. & Gupta R. K. Nuclear relaxation measurements of the geometry of enzyme-bound substrates and analogs. Methods. Enzymol. 49, 322–359 (1978). 651672 10.1016/s0076-6879(78)49017-2

[b22] Knowles J. R. Enzyme-Catalyzed Phosphoryl Transfer Reactions. Annu. Rev. Biochem. 49, 877–919 (1980). 6250450 10.1146/annurev.bi.49.070180.004305

[b23] Esser L. Synapsin I is structurally similar to ATP-utilizing enzymes. EMBO J. 17, 977–984 (1998). 9463376 10.1093/emboj/17.4.977 PMC1170447

[b24] Novak B. R., Moldovan D., Waldrop G. L. & de Queiroz M. S. Behavior of the ATP grasp domain of biotin carboxylase monomers and dimers studied using molecular dynamics simulations. Proteins 79, 622–632 (2010). 10.1002/prot.22910 21120858

[b25] Ye D. . Investigation of the Catalytic Site within the ATP-Grasp Domain of *Clostridium symbiosum* Pyruvate Phosphate Dikinase. J. Biol. Chem. 276, 37630–37639 (2001). 11468288 10.1074/jbc.M105631200

[b26] Yoshida M., Muneyuki E. & Hisabori T. ATP synthase—a marvellous rotary engine of the cell. Nat. Rev. Mol. Cell Biol. 2, 669–677 (2001). 11533724 10.1038/35089509

[b27] Yasuda R., Noji H., Yoshida M., Kinosita K. & Itoh H. Resolution of distinct rotational substeps by submillisecond kinetic analysis of F1-ATPase. Nature 410, 898–904 (2001). 11309608 10.1038/35073513

[b28] Kabaleeswaran V., Puri N., Walker J. E., Leslie A. G. W. & Mueller D. M. Novel features of the rotary catalytic mechanism revealed in the structure of yeast F1 ATPase. The EMBO Journal 25, 5433–5442 (2006). 17082766 10.1038/sj.emboj.7601410 PMC1636620

[b29] Adachi K. . Coupling of Rotation and Catalysis in F1-ATPase Revealed by Single-Molecule Imaging and Manipulation. Cell 130, 309–321 (2007). 17662945 10.1016/j.cell.2007.05.020

[b30] Shannon P. Cytoscape: A Software Environment for Integrated Models of Biomolecular Interaction Networks. Genome Res. 13, 2498–2504 (2003). 14597658 10.1101/gr.1239303 PMC403769

[b31] Doncheva N. T., Klein K., Domingues F. S. & Albrecht M. Analyzing and visualizing residue networks of protein structures. Trends Biochem. Sci. 36, 179–182 (2011). 21345680 10.1016/j.tibs.2011.01.002

[b32] Glaser F. . ConSurf: Identification of Functional Regions in Proteins by Surface-Mapping of Phylogenetic Information. Method. Biochem. Anal. 19, 163–164 (2003). 10.1093/bioinformatics/19.1.163 12499312

[b33] Ahmed A., Rippmann F., Barnickel G. & Gohlke H. A Normal Mode-Based Geometric Simulation Approach for Exploring Biologically Relevant Conformational Transitions in Proteins. J. Chem. Inf. Model. 51, 1604–1622 (2011). 21639141 10.1021/ci100461k

[b34] Howard J. Motor Proteins as Nanomachines: The Roles of Thermal Fluctuations in Generating Force and Motion. Biological Physics 47–59 (2010).

[b35] Feynman R., Leighton R., Sands M. & Hafner E. The Feynman Lectures on Physics; Vol. I, vol. 33 (AAPT, 1965).

[b36] Huxley A. F. A hypothesis for the mechanism of contraction of muscle. Prog Biophys Biophys Chem 7, 255–318 (1957). 13485191

[b37] Wang H. & Oster G. Ratchets, power strokes, and molecular motors. Appl. Phys. A 75, 315–323 (2002).

[b38] Eisenberg E. & Hill T. L. A cross-bridge model of muscle contraction. Prog. Biophys. Mol. Biol. 33, 55–82 (1979). 10.1016/0079-6107(79)90025-7 146885

[b39] Rosche E. & Westhoff P. Primary structure of pyruvate, orthophosphate dikinase in the dicotyledonous C 4 plant Flaveria trinervia. FEBS Lett. 273, 116–121 (1990). 2172023 10.1016/0014-5793(90)81064-u

[b40] Rosche E., Streubel M. & Westhoff P. Primary structure of the photosynthetic pyruvate orthophosphate dikinase of the C3 plant *Flaveria pringlei* and expression analysis of pyruvate orthophosphate dikinase sequences in C3, C3–C4 and C4 Flaveria species. Plant Mol. Biol. 26, 763–769 (1994). 7948930 10.1007/BF00013761

[b41] Salahas G., Manetas Y. & Gavalas N. Assaying for pyruvate, orthophosphate dikinase activity: necessary precautions with phosphoenolpyruvate carboxylase as coupling enzyme. Photosynth. Res. 24, 183–188 (1990). 24419911 10.1007/BF00032598

[b42] Kabsch, W. XDS. Acta Crystallogr. D. 66, 125–132 (2010). 20124692 10.1107/S0907444909047337 PMC2815665

[b43] Evans P. R. & Murshudov G. N. How good are my data and what is the resolution? Acta Crystallogr. D. 69, 1204–1214 (2013). 23793146 10.1107/S0907444913000061 PMC3689523

[b44] Collaborative, Computational Project and others. The CCP4 suite: programs for protein crystallography. Acta Crystallogr. D. 50, 760 (1994). 15299374 10.1107/S0907444994003112

[b45] McCoy A. J. . Phasercrystallographic software. J. Appl. Crystallogr. 40, 658–674 (2007). 19461840 10.1107/S0021889807021206 PMC2483472

[b46] Emsley P., Lohkamp B., Scott W. G. & Cowtan K. Features and development of Coot. Acta Crystallogr. D. 66, 486–501 (2010). 20383002 10.1107/S0907444910007493 PMC2852313

[b47] Adams P. D. . PHENIX : a comprehensive Python-based system for macromolecular structure solution. Acta Crystallogr. D. 66, 213–221 (2010). 20124702 10.1107/S0907444909052925 PMC2815670

[b48] Howlin B., Butler S. A., Moss D. S., Harris G. W. & Driessen H. P. C. TLSANL: TLS parameter-analysis program for segmented anisotropic refinement of macromolecular structures. J. Appl. Crystallogr. 26, 622–624 (1993).

[b49] Cowtan K. The Buccaneer software for automated model building. 1. Tracing protein chains. Acta Crystallogr. D. 62, 1002–1011 (2006). 16929101 10.1107/S0907444906022116

[b50] Murshudov G. N. . REFMAC 5 for the refinement of macromolecular crystal structures. Acta Crystallogr. D. 67, 355–367 (2011). 21460454 10.1107/S0907444911001314 PMC3069751

[b51] Afonine P. FEM.: Feature Enhanced Map. Acta Crystallogr. D. 71, 646–666 (2015). 25760612 10.1107/S1399004714028132 PMC4356370

[b52] Chen V. B. . MolProbity : all-atom structure validation for macromolecular crystallography. Acta Crystallogr. D. 66, 12–21 (2010). 20057044 10.1107/S0907444909042073 PMC2803126

[b53] Schrödinger LLC. The PyMOL Molecular Graphics System, Version 1.8 (2015).

[b54] Bernstein F. C. . The protein data bank: A computer-based archival file for macromolecular structures. J. Mol. Biol. 112, 535–542 (1977). 875032 10.1016/s0022-2836(77)80200-3

[b55] Pei J., Kim B.-H. & Grishin N. V. PROMALS3D: a tool for multiple protein sequence and structure alignments. Nucleic Acids Res. 36, 2295–2300 (2008). 18287115 10.1093/nar/gkn072 PMC2367709

[b56] Roe D. R. & Cheatham T. E. PTRAJ and CPPTRAJ: Software for Processing and Analysis of Molecular Dynamics Trajectory Data. J. Chem. Theory Comput. 9, 3084–3095 (2013). 26583988 10.1021/ct400341p

[b57] Case D. A. . The Amber biomolecular simulation programs. J. Comput. Chem. 26, 1668–1688 (2005). 16200636 10.1002/jcc.20290 PMC1989667

[b58] Roe D. R., Bergonzo C. & Cheatham T. E. Evaluation of Enhanced Sampling Provided by Accelerated Molecular Dynamics with Hamiltonian Replica Exchange Methods. J. Phys. Chem. B 118, 3543–3552 (2014). 24625009 10.1021/jp4125099 PMC3983400

[b59] Galindo-Murillo R., Roe D. R. & Cheatham T. E. Convergence and reproducibility in molecular dynamics simulations of the DNA duplex d(gcacgaacgaacgaacgc). Biochim. Biophys. Acta 1850, 1041–1058 (2015). 25219455 10.1016/j.bbagen.2014.09.007 PMC4339415

[b60] Hayward S. & Groot B. L. Normal Modes and Essential Dynamics. Molecular Modeling of Proteins 443, 89–106 (2008). 10.1007/978-1-59745-177-2_5 18446283

[b61] Ahmed A., Villinger S. & Gohlke H. Large-scale comparison of protein essential dynamics from molecular dynamics simulations and coarse-grained normal mode analyses. Proteins 78, 3341–3352 (2010). 20848551 10.1002/prot.22841

[b62] Katoh K. MAFFT: a novel method for rapid multiple sequence alignment based on fast Fourier transform. Nucleic Acids Res. 30, 3059–3066 (2002). 12136088 10.1093/nar/gkf436 PMC135756

[b63] Webb B. & Sali A. Comparative Protein Structure Modeling Using MODELLER. Current Protocols in Bioinformatics 54, 5.6.1–5.6.32 (2014). 10.1002/0471250953.bi0506s47 25199792

[b64] Word J., Lovell S. C., Richardson J. S. & Richardson D. C. Asparagine and glutamine: using hydrogen atom contacts in the choice of side-chain amide orientation. J. Mol. Biol. 285, 1735–1747 (1999). 9917408 10.1006/jmbi.1998.2401

[b65] Jorgensen W. L., Chandrasekhar J., Madura J. D., Impey R. W. & Klein M. L. Comparison of simple potential functions for simulating liquid water. J. Chem. Phys. 79, 926 (1983).

[b66] Hornak V. . Comparison of multiple Amber force fields and development of improved protein backbone parameters. Proteins 65, 712–725 (2006). 16981200 10.1002/prot.21123 PMC4805110

[b67] Homeyer N., Horn A. H. C., Lanig H. & Sticht H. AMBER force-field parameters for phosphorylated amino acids in different protonation states: phosphoserine, phosphothreonine, phosphotyrosine, and phosphohistidine. J. Mol. Model. 12, 281–289 (2005). 16240095 10.1007/s00894-005-0028-4

[b68] Ryckaert J.-P., Ciccotti G. & Berendsen H. J. Numerical integration of the cartesian equations of motion of a system with constraints: molecular dynamics of n-alkanes. J. Comput. Phys. 23, 327–341 (1977).

[b69] Cheatham T. E. I., Miller J. L., Fox T., Darden T. A. & Kollman P. A. Molecular Dynamics Simulations on Solvated Biomolecular Systems: The Particle Mesh Ewald Method Leads to Stable Trajectories of DNA, RNA, and Proteins. J. Am. Chem. Soc. 117, 4193–4194 (1995).

[b70] Berendsen H. J. C., Postma J. P. M., van Gunsteren W. F., DiNola A. & Haak J. R. Molecular dynamics with coupling to an external bath. J. Chem. Phys. 81, 3684 (1984).

[b71] Jacobs D. J., Rader A. J., Kuhn L. A. & Thorpe M. F. Protein flexibility predictions using graph theory. Proteins 44, 150–165 (2001). 11391777 10.1002/prot.1081

[b72] Torrie G. & Valleau J. Nonphysical sampling distributions in Monte Carlo free-energy estimation: Umbrella sampling. J. Comput. Phys. 23, 187–199 (1977).

[b73] Kumar S., Rosenberg J. M., Bouzida D., Swendsen R. H. & Kollman P. A. THE weighted histogram analysis method for free-energy calculations on biomolecules. I. The method. J. Comput. Chem. 13, 1011–1021 (1992).

