# Supplementary material for: Structural intermediates and directionality of the swiveling motion of Pyruvate Phosphate Dikinase
Source: Sci Rep. 2017 Mar 30;7:45389. doi: 10.1038/srep45389 (PMC5371819; doi:10.1038/srep45389)
Supplement: Supplementary Information [file srep45389-s1.pdf]

Supporting Information

Structural intermediates and directionality of the swiveling motion of Pyruvate Phosphate Dikinase

Alexander Minges[1,**], **Daniel Ciupka**[2,**], **Christian Winkler**[1], **Astrid Höppner**[1], **Holger Gohlke**[2,*] **and Georg Groth**[1,*]

[1]Cluster of Excellence on Plant Sciences (CEPLAS), Institute of Biochemical Plant Physiology, Heinrich Heine University Düsseldorf, 40204 Düsseldorf, Germany
[2]Institute of Pharmaceutical and Medicinal Chemistry, Heinrich Heine University Düsseldorf, 40204 Düsseldorf, Germany
[*]Correspondance should be addressed to H.G. (gohlke@hhu.de) or G.G (georg.groth@hhu.de).
[**]A.M. and D.C. contributed equally to this work.

January 30, 2017

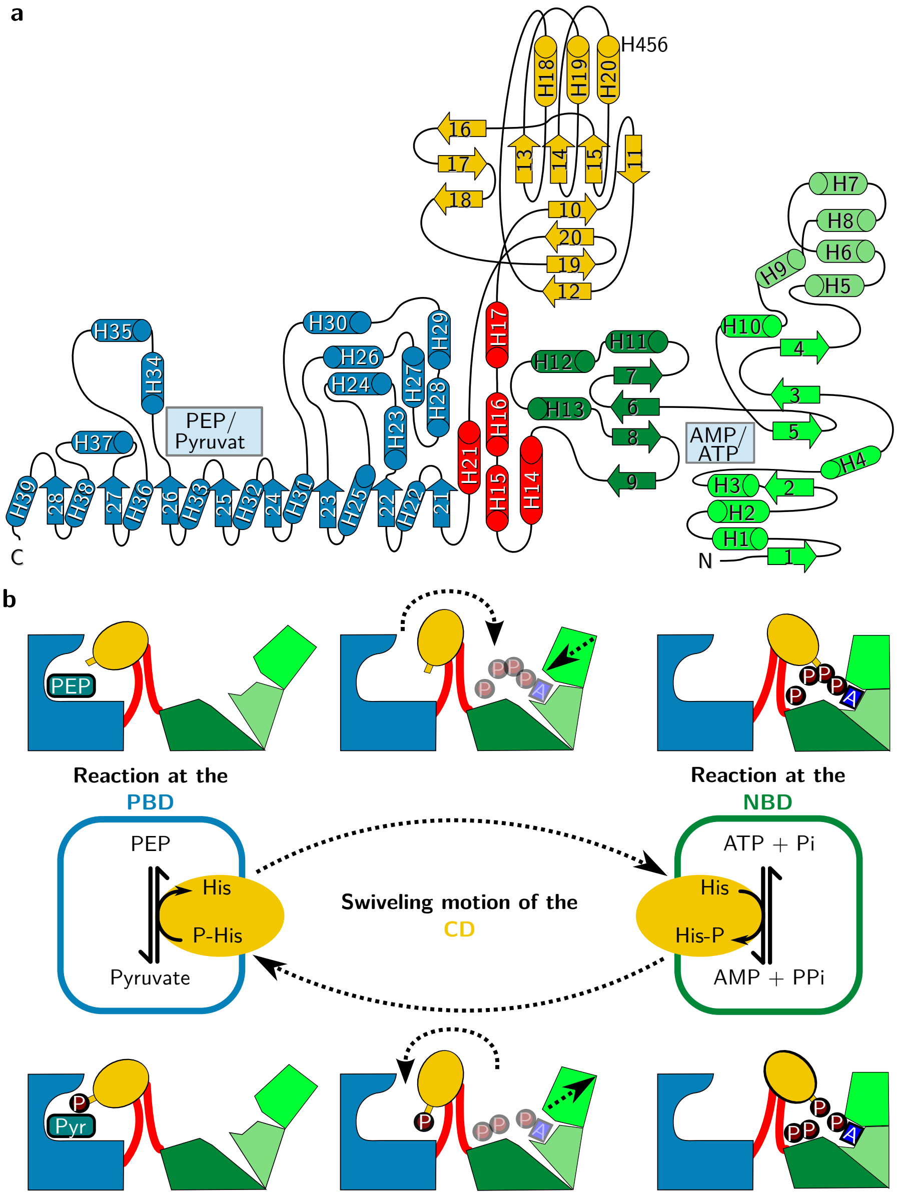

Figure 1 Topology diagram of PPDK and simplified model of the PPDK mechanism. (a) The central domain (CD, yellow) contains the catalytic histidine (H456) and is connected via the linker domain (LD, red) with the PEP/pyruvate-binding domain (PBD, colored blue), and nucleotide-binding domain (NBD, colored in three different greens according to the presence of three subdomains). The locations of the PEP/pyruvate and AMP/ATP binding sites are indicated. The secondary structure was predicted using DSSP [1]. (b) Simplified model of the PPDK swiveling domain mechanism, adapted from refs. [2, 3]. The PPDK-catalyzed reaction (middle row) involves a phosphoryl transfer via H456 of the CD (yellow) between the locations of the two reactions at the NBD (green, right side) and at the PBD (blue, left side). The dotted arrows in the cartoons in the top and bottom rows indicate motions of the CD and within the NBD.

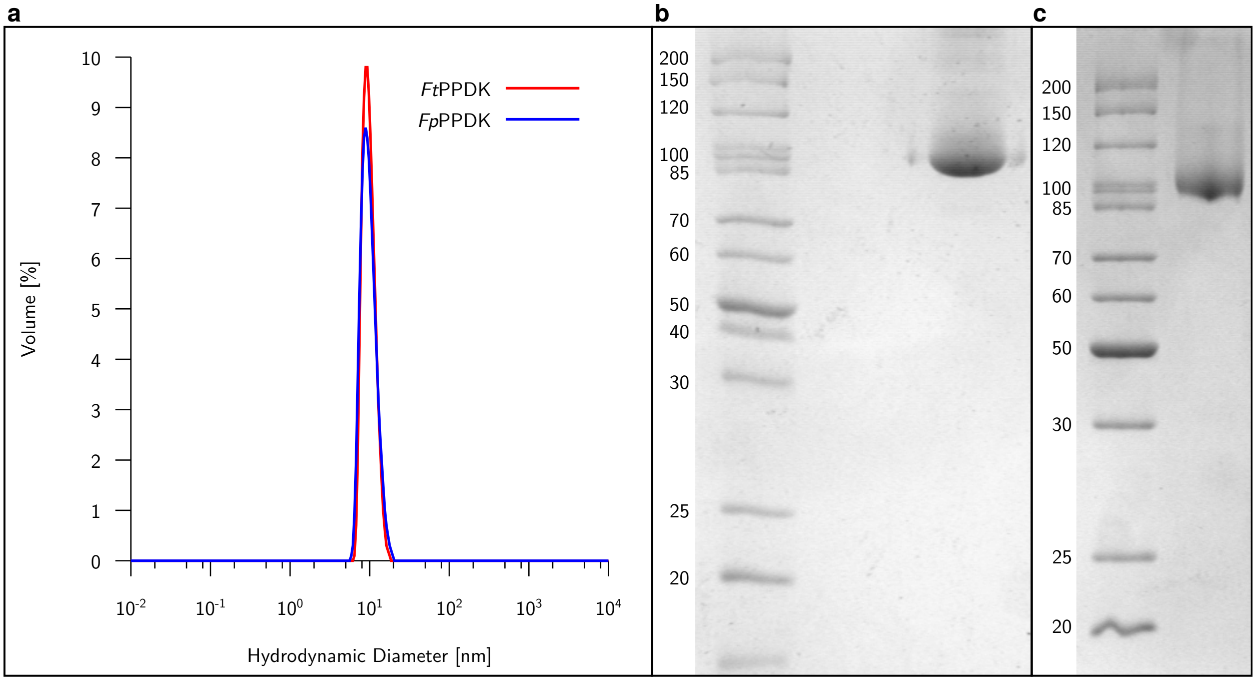

Figure 2 Quality assessment of PPDK purification. (a) Size distribution analysis of triplicate dynamic light scattering (DLS) measurements of *Ft*PPDK and *Fp*PPDK prior to crystallization. In both cases, DLS confirms the monodispersity of the sample. The mean hydrodynamic diameter of *Ft*PPDK (red) is 10.72±2.17 nm corresponding to an estimated molecular weight of 171.0±35.8 kDa. For *Fp*PPDK (blue), the mean hydrodynamic diameter was determined to be 10.72±2.79 nm with a corresponding molecular weight of 171.0±49.3 kDa. (b/c) Colloidal Coomassie-stained SDS PAGE of purified PPDK. *Fp*PPDK (b) and *Ft*PPDK (c) are visible as prominent bands at approx. 100 kDa.

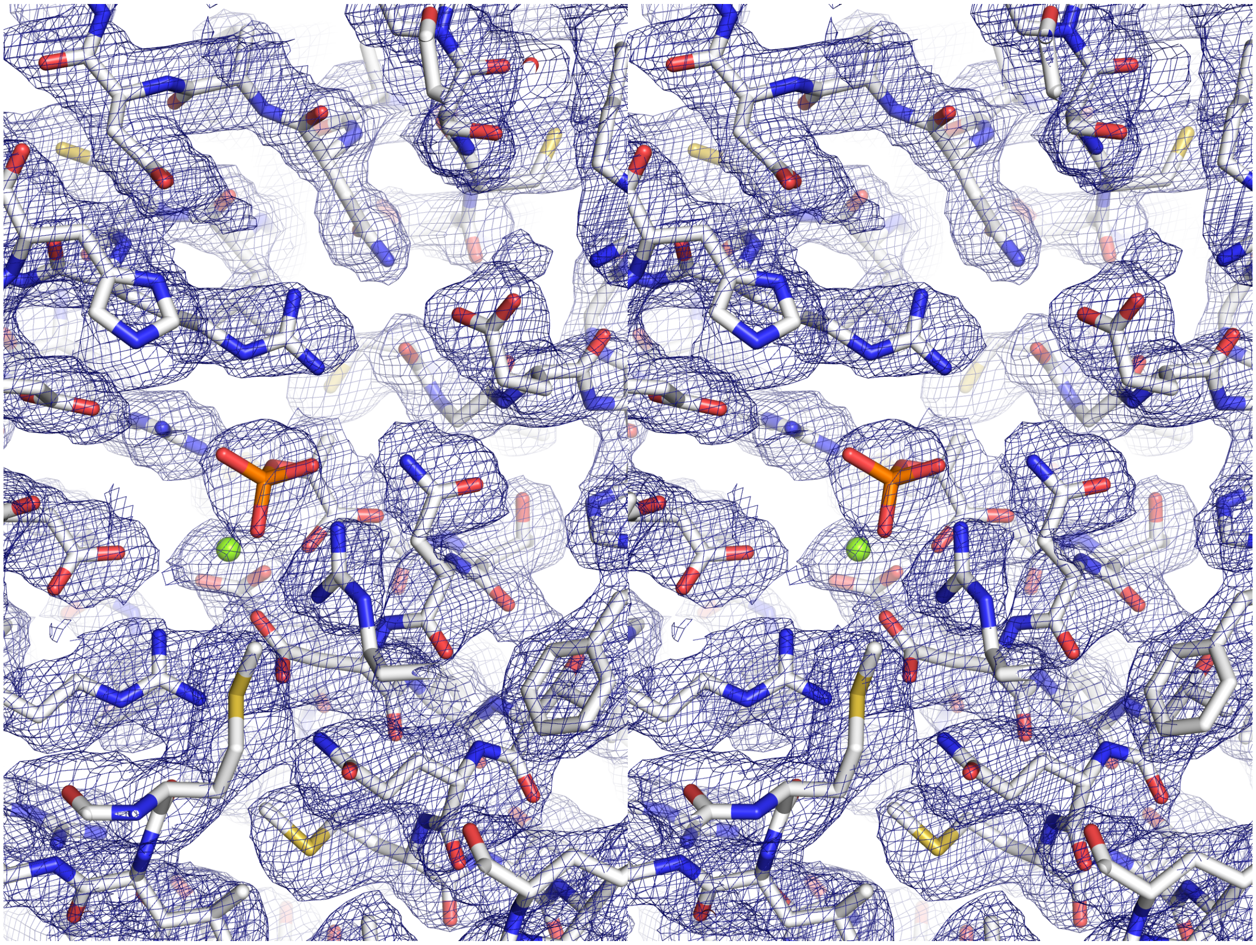

Figure 3 Electron density of PEP binding site in *Ft*PPDK. Stereo image illustrating the quality of electron density around bound PEP in 5JVL/A. Maps shown are feature-enhanced maps (FEM) [4] contoured at the equivalent of 1.0 σ.

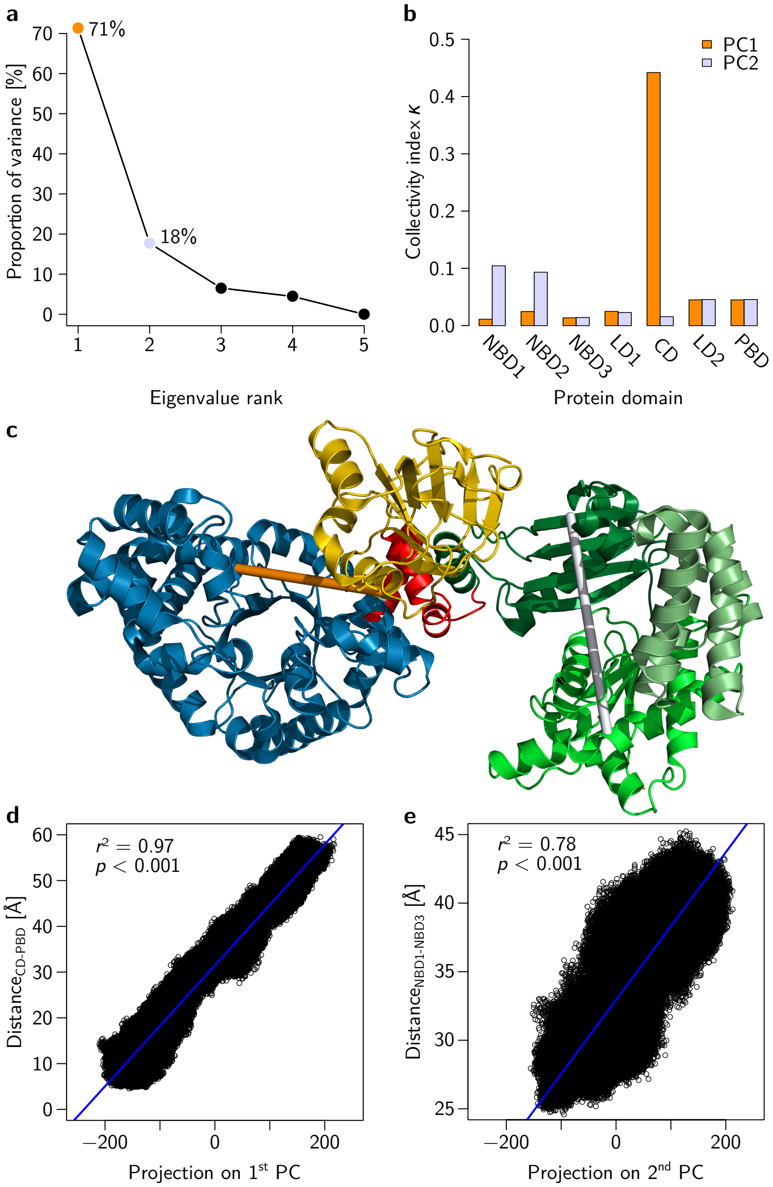

Figure 4 Principal component analysis on PPDK and reaction coordinates for PMF calculation. (a) The proportion of explained variance by each principal component ranked by its eigenvalue. The first (gold) and second (silver) principal component (PC) relate to Fig. 4b. 89% of the variance can be explained by the first two PCs. (b) Domain-wise collectivity index κ of the first and second PC, calculated according to [5]. (c) The distance$_{\text{CD-PBD}}$ (depicted as gold line) and distance$_{\text{NBD1-NBD3}}$ (depicted as silver line) between $\text{H456}_{C_\alpha} - \text{H565}_{C_\alpha}$ and $\text{S215}_{C_\alpha} - \text{E272}_{C_\alpha}$ (numbering according to *F. trinervia / pringlei*) are used as reaction coordinates for the swiveling motion of the CD and the opening-closing motion of the NBD, respectively. The depicted structure was taken from PDB ID 5JVN. The domain coloring is according to Fig. 1a. (d/e) Validation of the reaction coordinates to represent the swiveling motion of the CD and the opening-closing motion of the NBD. Scatter plots of the distances $\text{H456}_{C_\alpha} - \text{H565}_{C_\alpha}$ (e) and $\text{S215}_{C_\alpha} - \text{E272}_{C_\alpha}$ (e) versus projections onto the first (d) and second (e) principle component obtained from the PCA over the cluster representatives (Fig. 4b); each dot represents one conformation generated by MD simulations of ~10 µs length. The correlation line is shown in blue.

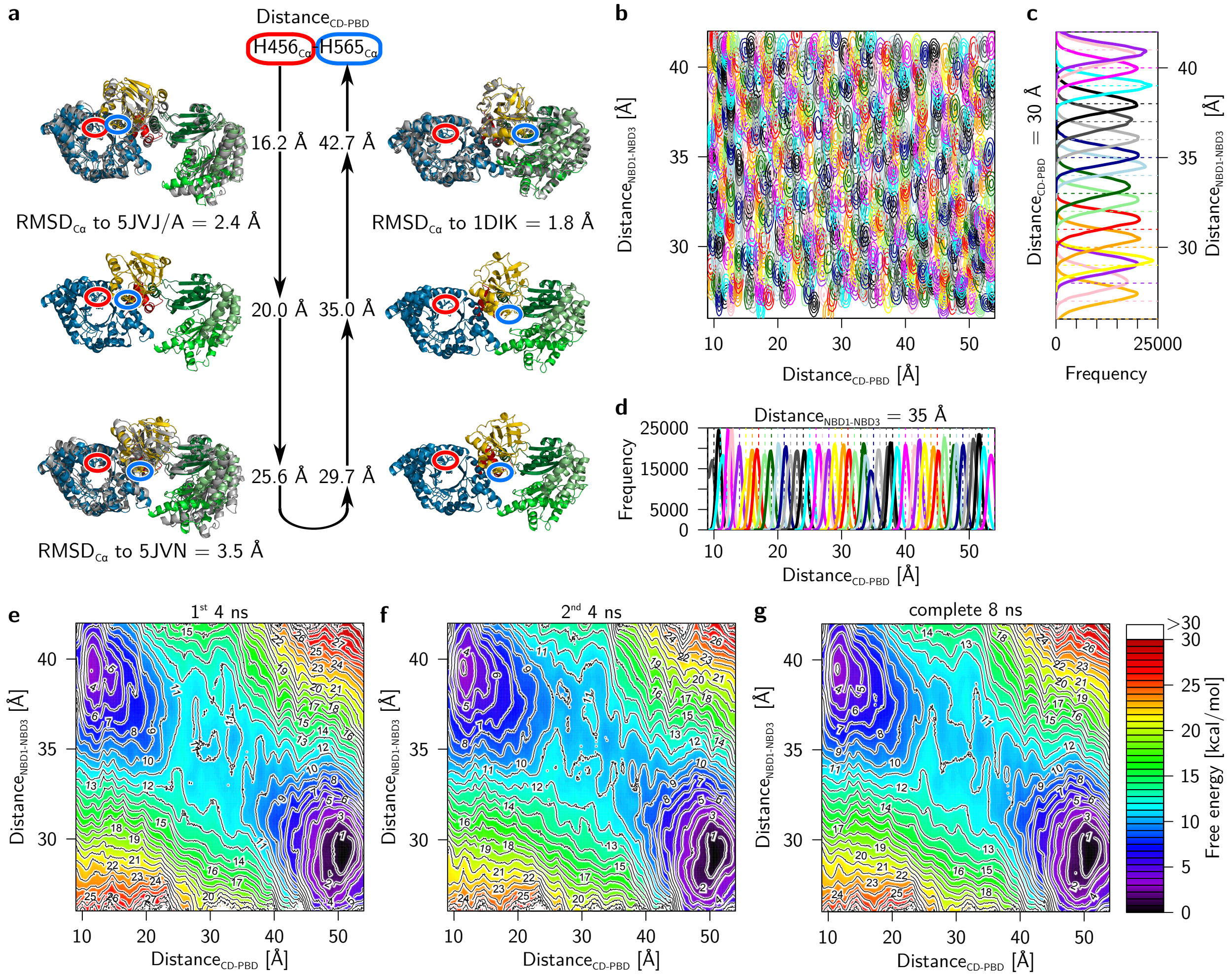

Figure 5 Transition pathway for and validation of PMF calculations. Transition pathway between conformations III and I (Fig. 5b in the main text), created by targeted simulations with NMSim [5] starting from homology models with the template structure PDB ID 1KBL and 1KC7 towards homology models with the template structure PDB ID 1VBH and 1VBG. Intermediate conformations are shown at approximately 5 Å spacing along the reaction coordinate distance$_{\text{CD-PBD}}$ (Fig. 4c) (depicted next to the conformations). The generated conformations are overlaid with cluster representatives of PPDK crystal structures (depicted in gray), and the C_α-atom RMSD is given. (b-d) Overlap of umbrella sampling simulations of non-phosphroylated *Ft*PPDK. Frequency distributions of the sampled distances used as reaction coordinates (Fig. 4c) for umbrella sampling are shown (b) for the 782 MD simulations covering the 2D space spanned by both reaction coordinates (contour lines are plotted at frequencies of 5000, 10000, 15000, 20000, and 25000 conformations) and (c) for 17 MD simulations at a restrained distance$_{\text{CDPBD}}$ = 30 Å as well as (d) for 45 MD simulations at a restrained distance$_{\text{NBD1NBD3}}$ = 35 Å. Each color represents one MD simulation. (e-g) Convergence of the 2D PMF calculation of phosphorylated *Ft*PPDK. 2D PMFs computed using umbrella sampling along the two reaction coordinates (Fig. 4c) are shown for (e) only the 1st half of the sampling time, (f) the 2nd half, and (g) the complete sampling time of 8 ns per window.

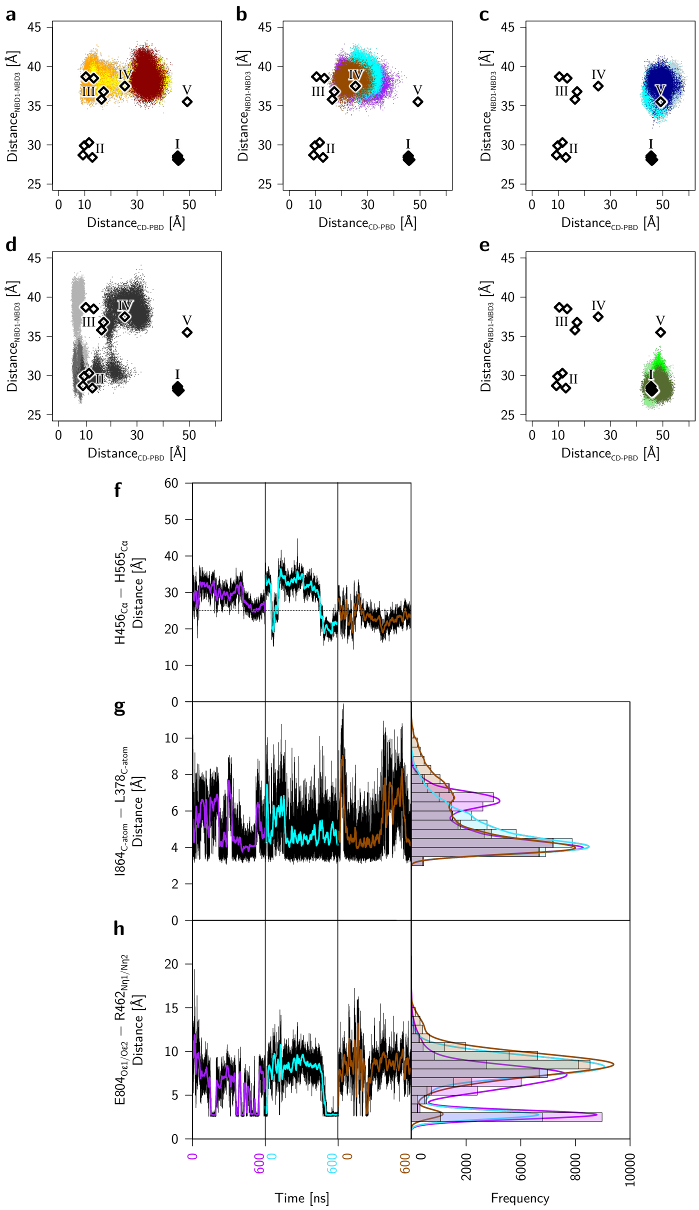

Figure 6 Unrestrained MD simulations of PPDK. (a-e) Projection of conformations from MD simulations and crystal structures of PPDK (dots; each color represents one MD simulation of non-phosphorylated *Ft*PPDK, see Tab. S3) (using the corresponding residues for each organism (Tab. S1), with Roman numerals according to Fig. 4a) onto the reaction coordinates distance$_{\text{CD-PBD}}$ and distance$_{\text{NBD1-NBD3}}$ (Fig. 4c). (I: PDB ID 1DIK, 2DIK, 1GGO, 1JDE, 1KBL, 1KC7; II: 5JVJ/B, 5JVL/A, 5JVL/C, 5JVL/D; III: 2R82, 1VBG, 1VBH, 5JVJ/A; IV: 5JVN; V 2X0S). Three MD simulations of 600 ns length each were started from (a) conformation III, (b) conformation IV, (c) conformation V (d) conformation II, and (e) conformation I. (f-h) Interactions observed in three MD simulations of 600 ns length each started from conformation IV. (f) The swiveling motion of the CD is analzed by the distance$_{\text{CD-PBD}}$ between H456$_{C_\alpha}$ – H565$_{C_\alpha}$, (g) a non-polar interaction analyzed by the minimal distance between all C-atoms of I864 and L378, and (h) a salt bridge is analyzed by the minimal distance between atoms E804$_{O\varepsilon1/O\varepsilon2}$ and R462$_{N\eta1/N\eta2}$.

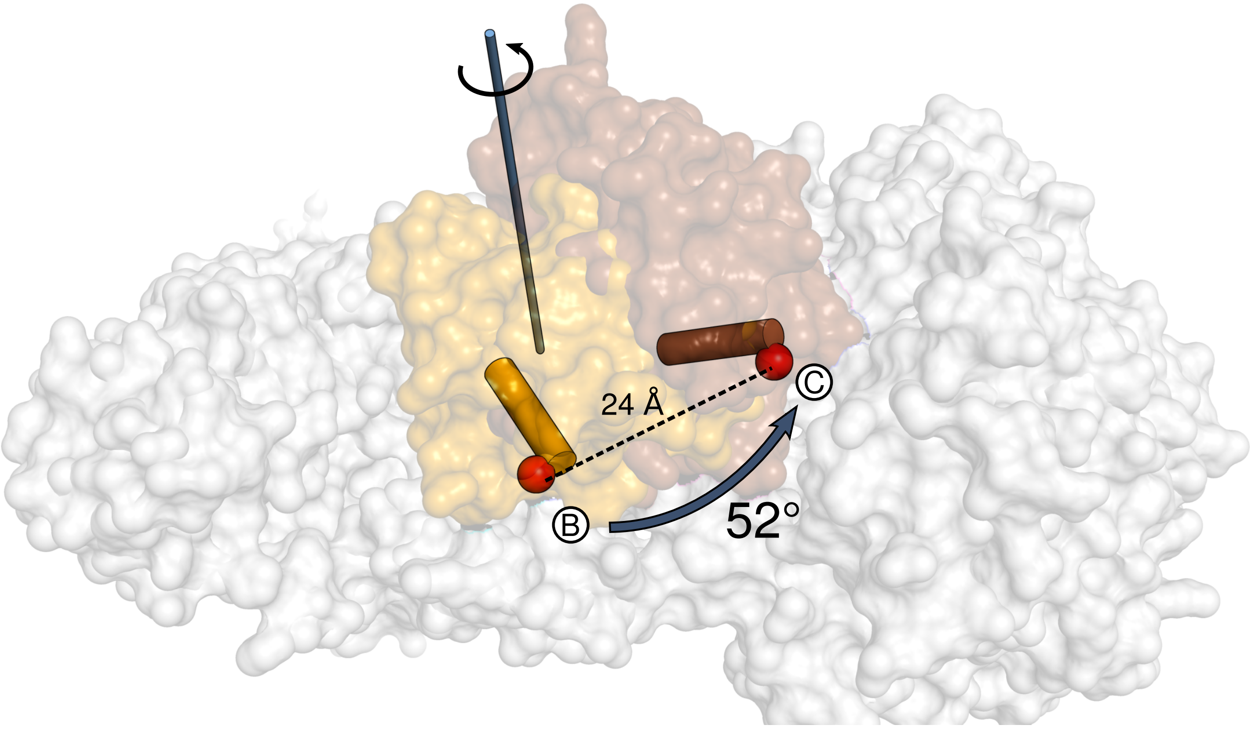

Figure 7 Stepped movement of the CD. CDs of 5JVN (B, yellow) and 1KBL (C, brown). Helix 20 containing the catalytic H456 (H455 in 1KBL) is depicted as cylinder with the C_α atom of H456 shown as red sphere. The rotational axis for the transitions between states B and C is depicted as a blue arrow. The distance between the C_α atoms of the catalytic histidine is shown as a dashed line.

Table 1 Overview of all currently known PPDK structures*

Cluster†	Organism	PDB ID	Comment	$d_{CD\text{-}PBD}$§ (Å)	$d_{NBD1\text{-}NBD3}$¶ (Å)	Reference
I	*C. symbiosum*	1DIK		45.5	28.1	[2]
I	*C. symbiosum*	2DIK	R337A	45.8	28.0	[3]
I	*C. symbiosum*	1GGO	T453A	45.5	28.6	[6]
I	*C. symbiosum*	1JDE	K22A	46.2	28.1	[7]
I	*C. symbiosum*	1KBL		45.6	28.6	[8]
I	*C. symbiosum*	1KC7	PEP analogue bound	45.4	28.4	[8]
**II**	***F. trinervia***	**5JVJ**	**Chain B; PEP bound; CD and NBD incomplete**	**11.6**	**30.3**	**This paper**
**II**	***F. trinervia***	**5JVL**	**Chain A; PEP and ATP analogue bound**	**12.9**	**28.4**	**This paper**
**II**	***F. trinervia***	**5JVL**	**Chain C; PEP and ATP analogue bound**	**9.8**	**29.9**	**This paper**
**II**	***F. trinervia***	**5JVL**	**Chain D; PEP and ATP analogue bound**	**9.3**	**28.7**	**This paper**
III	*C. symbiosum*	2R82	R219E/E271R/S262D	10.4	38.7	[8]
III	*Z. mays*	1VBG		16.4	35.8	[9]
III	*Z. mays*	1VBH	PEP bound	17.2	36.8	[9]
**III**	***F. trinervia***	**5JVJ**	**Chain A; PEP bound**	**13.4**	**38.5**	**This paper**
**IV**	***F. pringlei***	**5JVN**	**PEP and ATP analogue bound**	**25.3**	**37.5**	**This paper**
V	*T. brucei*	2X0S		49.2	35.5	[10]
**‡**	***F. trinervia***	**5JVL**	**Chain B; PEP bound; NBD not resolved**	**15.5**	**-**	**This paper**
‡	*C. symbiosum*	2FM4	NMR-derived structure of the CD	-	-	[11]

*Crystal structures described in this work are shown in bold.

†The cluster number relates to Fig. 4A. Within each cluster, structures are sorted in chronological order.

‡Structure not used for analysis; due to missing domains.

§Measured between C_α atoms of residues 455-564 (*C. symbiosum*); 456-565 (*F. trinervia*); 456-565 (*F. pringlei*); 482-589 (*T. brucei*); 458-567 (*Z. mays*).

¶Measured between C_α atoms of residues 214-271 (*C. symbiosum*); 215-272 (*F. trinervia*); 215-272 (*F. pringlei*); 218-275 (*T. brucei*); 217-274 (*Z. mays*).

Table 2 Structural comparison of PPDK crystal structures by 2D C_α-atom RMSD (Å)*

	Cluster I†						Cluster II†				Cluster III†				Cluster IV†	Cluster V†
	1DIK	2DIK	1GGO	1JDE	1KBL	1KC7	5JVJ/B	5JVL/A	5JVL/C	5JVL/D	2R82	1VBG	1VBH	5JVJ/A	5JVN	2X0S
1DIK‡	0	0.4	0.5	0.6	0.9	0.9	10.7	9.2	10.8	10.6	11	10	9.9	10.9	8.1	5.9
2DIK	0.4	0	0.5	0.6	1	1	10.7	9.2	10.8	10.6	11	10	9.9	10.9	8.1	5.8
1GGO	0.5	0.5	0	0.7	0.9	0.9	10.6	9.2	10.7	10.5	10.9	9.9	9.8	10.8	8	5.8
1JDE	0.6	0.6	0.7	0	1.1	1.1	10.7	9.2	10.8	10.6	11	10	9.9	10.9	8.1	5.9
1KBL	0.9	1	0.9	1.1	0	0.3	10.7	9.2	10.7	10.6	10.9	10	9.9	10.8	8	5.8
1KC7	0.9	1	0.9	1.1	0.3	0	10.7	9.2	10.7	10.5	11	10	9.9	10.9	8	5.9
5JVJ/B	10.7	10.7	10.6	10.7	10.7	10.7	0	3.2	1.5	2.7	5.1	4.5	5	4.6	6.1	11.4
5JVL/A	9.2	9.2	9.2	9.2	9.2	9.2	3.2	0	2.6	2.2	5	4.4	4.7	5.2	5.6	10.8
5JVL/C‡	10.8	10.8	10.7	10.8	10.7	10.7	1.5	2.6	0	1.4	5.1	4.8	5.3	5	6.4	11.8
5JVL/D	10.6	10.6	10.5	10.6	10.6	10.5	2.7	2.2	1.4	0	5.3	5.3	5.7	5.7	6.7	12
2R82	11	11	10.9	11	10.9	11	5.1	5	5.1	5.3	0	3	3.1	2.9	5.5	10.8
1VBG‡	10	10	9.9	10	10	10	4.5	4.4	4.8	5.3	3	0	0.8	2.2	4.6	9.7
1VBH	9.9	9.9	9.8	9.9	9.9	9.9	5	4.7	5.3	5.7	3.1	0.8	0	2.4	4.5	9.5
5JVJ/A	10.9	10.9	10.8	10.9	10.8	10.9	4.6	5.2	5	5.7	2.9	2.2	2.4	0	4.5	10.3
5JVN‡	8.1	8.1	8	8.1	8	8	6.1	5.6	6.4	6.7	5.5	4.6	4.5	4.5	0	7.5
2X0S‡	5.9	5.8	5.8	5.9	5.8	5.9	11.4	10.8	11.8	12	10.8	9.7	9.5	10.3	7.5	0

*The 2D $C_\alpha$atom RMSD was computed over all C_α atoms common and resolved in all PPDK structures with all domain resolved. Small RMSD are highlighted by blue, high RMSD by orange.

†The clusters relate to Fig. 4a and are characterized by a closed NBD combined with the CD facing the NBD (cluster I), a closed NBD with the CD-facing the PBD (cluster II), an open NBD with the CD facing the PBD (cluster III), an open NBD with the CD in between the PBD and NBD (cluster IV), and an open NBD with the CD facing the NBD (cluster V).

‡Cluster representatives

Table 3 Unrestrained MD simulations of non-phosphorylated *Ft*PPDK*

Starting conformation	Repetition	Simulation length (ns)	Color in figures
I	I.1	600	Light green
I	I.2	600	Green
I	I.3	600	Dark olivegreen
II	II.1	600	Light gray
II	II.2	600	Gray
II	II.3	600	Dark gray
III	III.1	600	Yellow
III	III.2	600	Orange
III	III.3	1500	Dark red
Started from III.3 at 800 ns	III.4	700	Dark orange
IV	IV.1	600	Cyan
IV	IV.2	600	Purple
IV	IV.3	600	Brown
V	V.1	600	Light blue
V	V.2	600	Dark cyan
V	V.3	600	Dark blue

*Three replicate simulations were started from each cluster, using crystal structures of *Ft*PPDK, if available at the beginning of this project, or homology models (see Supplemental Methods).

References

[1] Kabsch, W. & Sander, C. Dictionary of protein secondary structure: Pattern recognition of hydrogen-bonded and geometrical features. *Biopolymers* **22**, 2577–2637 (1983).

[2] Herzberg, O. *et al.* Swiveling-domain mechanism for enzymatic phosphotransfer between remote reaction sites. *P. Natl. Acad. Sci. USA.* **93**, 2652–2657 (1996).

[3] McGuire, M. *et al.* Determination of the Nucleotide Binding Site within Clostridium symbiosum Pyruvate Phosphate Dikinase by Photoaffinity Labeling, Site-Directed Mutagenesis, and Structural Analysis. *Biochemistry-US.* **35**, 8544–8552 (1996).

[4] Afonine, P. FEM: Feature Enhanced Map. *Acta Crystallogr. D.* **71**, 646–666 (2015).

[5] Ahmed, A., Rippmann, F., Barnickel, G. & Gohlke, H. A Normal Mode-Based Geometric Simulation Approach for Exploring Biologically Relevant Conformational Transitions in Proteins. *J. Chem. Inf. Model.* **51**, 1604–1622 (2011).

[6] Wei, M., Li, Z., Ye, D., Herzberg, O. & Dunaway-Mariano, D. Identification of Domain-Domain Docking Sites within Clostridium symbiosum Pyruvate Phosphate Dikinase by Amino Acid Replacement. *J. Biol. Chem.* **275**, 41156–41165 (2000).

[7] Ye, D. *et al.* Investigation of the Catalytic Site within the ATP-Grasp Domain of Clostridium symbiosum Pyruvate Phosphate Dikinase. *J. Biol. Chem.* **276**, 37630–37639 (2001).

[8] Herzberg, O. *et al.* Pyruvate site of pyruvate phosphate dikinase: crystal structure of the enzyme-phosphonopyruvate complex, and mutant analysis. *Biochemistry-US.* **41**, 780–787 (2002).

[9] Nakanishi, T., Nakatsu, T., Matsuoka, M., Sakata, K. & Kato, H. Crystal Structures of Pyruvate Phosphate Dikinase from Maize Revealed an Alternative Conformation in the Swiveling-Domain Motion. *Biochemistry-US.* **44**, 1136–1144 (2005).

[10] Cosenza, L. W., Bringaud, F., Baltz, T. & Vellieux, F. M. The 3.0Å Resolution Crystal Structure of Glycosomal Pyruvate Phosphate Dikinase from Trypanosoma brucei. *J. Mol. Biol.* **318**, 1417–1432 (2002).

[11] Lin, Y., Lusin, J. D., Ye, D., Dunaway-Mariano, D. & Ames, J. B. Examination of the Structure, Stability, and Catalytic Potential in the Engineered Phosphoryl Carrier Domain of Pyruvate Phosphate Dikinase. *Biochemistry-US.* **45**, 1702–1711 (2006).
